# SUVmax-IPI as a New Prognostic Index in Metastatic Non-Small Cell Lung Cancer Patients Receiving Nivolumab

**DOI:** 10.3390/curroncol32100566

**Published:** 2025-10-09

**Authors:** Nagihan Kolkıran, Atike Pınar Erdoğan, Mustafa Şahbazlar, Semra Taş, Gamze Gököz Doğu, Kübra Canaslan, İlkay Tuğba Ünek, Özge Demirkıran, Bilgin Demir, Güler Nur Teküstün, Özgür Tanrıverdi, Ferhat Ekinci

**Affiliations:** 1 Department of Medical Oncology, Faculty of Medicine, Manisa Celal Bayar University, Manisa 45030, Turkey; atike.erdogan@cbu.edu.tr (A.P.E.); mustafa.sahbazlar@cbu.edu.tr (M.Ş.); ekinci.ferhat@cbu.edu.tr (F.E.); 2 Department of Medical Oncology, Faculty of Medicine, Pamukkale University, Denizli 20070, Turkey; stas@pau.edu.tr (S.T.); ggokoz@pau.edu.tr (G.G.D.); 3 Department of Medical Oncology, Faculty of Medicine, Dokuz Eylül University, İzmir 35330, Turkey; kubra.canaslan@deu.edu.tr (K.C.); tugba.gun@deu.edu.tr (İ.T.Ü.); 4 Department of Medical Oncology, Faculty of Medicine, Aydın Adnan Menderes University, Aydın 09100, Turkey; ozgedemirkiran@adu.edu.tr (Ö.D.); bdemir@adu.edu.tr (B.D.); 5 Department of Medical Oncology, Faculty of Medicine, Muğla Sıtkı Koçman University, Muğla 48000, Turkey; gulernurceltik@gmail.com (G.N.T.); dr.ozgur.tanriverdi@gmail.com (Ö.T.)

**Keywords:** nivolumab, inflammatory markers, non-small cell lung cancer, survival outcomes

## Abstract

**Simple Summary:**

Despite the success of immunotherapy with nivolumab in advanced non-small cell lung cancer, many patients still experience poor outcomes. Reliable tools are needed to better predict which patients are most likely to benefit from treatment. This study introduces SUVmax-IPI, a novel and practical biomarker that combines two routinely assessed parameters: tumor metabolic activity and systemic inflammation. It aimed to predict survival outcomes in patients with metastatic non-small cell lung cancer receiving nivolumab. By integrating positron emission tomography imaging and standard laboratory tests, this score provides a simple and cost-effective tool for clinicians to better identify patients at higher risk of poor outcomes. SUVmax-IPI can help guide treatment decisions, improve patient counseling, and enhance personalized care in the immunotherapy setting.

**Abstract:**

Background/Objectives: Nivolumab has significantly improved outcomes in patients with metastatic non-small cell lung cancer (NSCLC); however, reliable prognostic biomarkers remain an unmet need. To address this gap, we developed the SUVmax-IPI, a novel prognostic index combining maximum standardized uptake value (SUVmax) from ^18^F-fluorodeoxyglucose positron emission tomography (FDG-PET) with systemic inflammatory markers. This study aimed to evaluate the prognostic value of SUVmax-IPI in patients with NSCLC receiving nivolumab therapy. Methods: This multicenter retrospective analysis included 187 patients with metastatic NSCLC receiving nivolumab across 5 tertiary institutions. The SUVmax-IPI incorporated pretreatment SUVmax and laboratory-based inflammatory prognostic index (IPI) parameters. Survival outcomes were evaluated using Kaplan–Meier analysis with log-rank testing and multivariate cox regression. Results: Receiver operating characteristic (ROC) analysis established an optimal SUVmax-IPI cut-off of 241.9. Patients with SUVmax-IPI ≤ 241.9 had significantly better survival outcomes: median overall survival (OS) was 35 versus 15 months (*p* = 0.002). For progression-free survival (PFS), although a numerical difference favored patients with SUVmax-IPI ≤ 241.9 (median: 15 vs. 8 months), this did not reach statistical significance (log-rank *p* = 0.175). Multivariate analysis confirmed SUVmax-IPI as an independent predictor of survival (*p* = 0.002). Conclusions: The SUVmax-IPI represents a promising prognostic tool for patients with metastatic NSCLC who received at least 3 months of nivolumab, integrating metabolic and inflammatory parameters to predict survival outcomes.

## 1. Introduction

Non-small cell lung cancer continues to dominate global cancer mortality, with metastatic disease accounting for more than 60% of diagnoses and a 5-year survival rate below 10% [1]. The advent of immune checkpoint inhibitors, particularly nivolumab, has redefined therapeutic paradigms, demonstrating superior survival outcomes compared to chemotherapy in both squamous and non-squamous NSCLC subtypes [2,3]. However, durable responses are not achieved in all cases, with intrinsic resistance observed in a significant subset, highlighting the urgent need for reliable prognostic biomarkers [4]. While programmed death ligand 1 (PD-L1) expression and tumor mutational burden are established predictive markers, their clinical utility is limited by spatial heterogeneity, assay variability, and dynamic changes during therapy [4].

Metabolic dysregulation is a central feature of oncogenesis, characterized by a pronounced shift toward aerobic glycolysis (Warburg effect) [5]. This adaptive mechanism drives excessive glucose consumption even under oxygen-rich conditions, enabling tumor survival and proliferation across diverse malignancies [5]. FDG-PET provides a non-invasive means to quantify this glycolytic phenotype, with tumor SUVmax reflecting glucose transporter (GLUT1) expression and hexokinase activity—key mediators of metabolic reprogramming in NSCLC [6]. Recent studies have demonstrated that metabolic parameters such as SUVmax, metabolic tumor volume, and total lesion glycolysis can predict responses to immune checkpoint inhibitors, with elevated baseline SUVmax values associated with less favorable outcomes [7,8].

In addition to tumor-intrinsic metabolic pathways, emerging evidence highlights the critical role of host systemic inflammation in modulating immunotherapy outcomes, with hematologic indices and biochemical markers serving as quantifiable prognostic indicators. The Lung Immune Prognostic Index (LIPI), which integrates derived neutrophil-to-lymphocyte ratio (dNLR) and elevated lactate dehydrogenase, stratifies patients with metastatic NSCLC into distinct survival outcomes [9]. Similarly, pretreatment systemic immune-inflammation index (SII), neutrophil-to-lymphocyte ratio (NLR), and platelet-to-lymphocyte ratio (PLR) were independently associated with reduced progression-free survival and overall survival in those receiving nivolumab [10].

In the context of early-stage disease, Tomita and colleagues demonstrated the validity of the IPI—a composite biomarker incorporating C-reactive protein (CRP), NLR, and serum albumin levels—as a multidimensional predictor of survival [11].

To further advance this field, we developed the SUVmax-IPI as a new prognostic tool that integrates tumor metabolic activity measured by FDG-derived SUVmax with the systemic inflammatory evaluation provided by the IPI.

This study aimed to evaluate the prognostic value of the SUVmax-IPI in patients with metastatic NSCLC receiving nivolumab treatment. We hypothesized that this combined assessment of tumor glycolytic phenotype and host inflammatory status would improve prognostic stratification beyond conventional markers.

## 2. Materials and Methods

This retrospective multicenter study was conducted across 5 tertiary care centers in Turkey: Manisa Celal Bayar University, Pamukkale University, Dokuz Eylül University, Aydın Adnan Menderes University, and Muğla Sıtkı Koçman University Hospitals. The study cohort included 187 consecutive patients with metastatic NSCLC and who were treated with nivolumab between May 2019 and December 2024. Eligible patients were ≥18 years with histologically confirmed metastatic NSCLC. Since a 12-week interval was required for response evaluation, inclusion also required receipt of ≥3 months of nivolumab and availability of a post-treatment FDG-PET. Patients with nivolumab treatment duration less than three months or without posttreatment FDG-PET or laboratory data were excluded. Clinical, pathological, radiological, and treatment-related variables were retrospectively collected from electronic medical records. The analyzed parameters included demographic data (age at diagnosis, sex, smoking history), tumor characteristics (histologic subtype, mutation status, PD-L1 expression level, distribution of metastatic lesions), and treatment history (prior systemic therapies including regimen type and line of therapy, as well as nivolumab-specific details such as treatment line and duration). Mutation status was evaluated using next-generation sequencing panels, and the analysis considered the presence of any actionable mutation (including but not limited to EGFR, ALK, ROS1). PD-L1 expression was assessed using the tumor proportion score. A cut-off value of ≥1% was considered positive. PD-L1 was evaluated using the VENTANA PD-L1 (SP263) Assay with the OptiView DAB IHC Detection Kit on the BenchMark ULTRA automated platform (Roche Tissue Diagnostics, Tucson, AZ, USA). Clinical outcomes were assessed through radiographic response evaluation according to RECIST v1.1 criteria, documentation of immune-related adverse events, and survival outcomes including PFS and OS.

FDG-PET acquisition and analysis followed a harmonized, standardized protocol across all five sites, in accordance with institutional procedures and international guidelines. Patients fasted for 6 h and proceeded only if blood glucose was <200 mg/dL. Imaging was performed on a General Electric Discovery IQ 3-Ring FDG-PET (GE Healthcare, Milwaukee, WI, USA). A low-dose Computed Tomography (16-slice; 120 kVp) was obtained for attenuation correction and anatomical localization, followed by PET from mid-thigh to skull base. Lesions were delineated semi-automatically using Volume Computer-Assisted Reading with a 41% SUVmax isocontour. Metabolically active lesions were segmented semi-automatically with FDG-PET Volume Computer-Assisted Reading, applying a 41% SUVmax isocontour to define the volume of interest. To minimize inter-site variability, acquisition, reconstruction, review workflow, and lesion-segmentation rules were enforced via written standard operating procedures and routine quality control.

For quantitative biomarker assessment, SUVmax values were obtained from the most metabolically active lesion identified on pretreatment FDG-PET imaging. The IPI was calculated using the formula: IPI = (CRP × NLR)/albumin. Building on this, a new prognostic score—SUVmax-IPI—was derived as follows: SUVmax-IPI = SUVmax × IPI. ROC curve analysis, applying Youden’s index (J = 0.1824), identified 241.9 as the optimal cut-off value for predicting clinical outcomes. The definitions and calculation formulas of variables are summarized in Supplementary Table S1, and the raw input data—including the final SUVmax-IPI values—are provided in Supplementary Table S2.

Descriptive statistics for categorical variables are reported as frequencies and percentages while continuous variables are summarized as medians with interquartile ranges (IQRs). Survival outcomes including PFS and OS were evaluated using Kaplan–Meier analysis, with between-group comparisons performed via the log-rank test. Univariable Cox proportional hazards regression identified potential prognostic factors, and were subsequently analyzed in multivariable Cox regression analyses. Results are presented as adjusted hazard ratios (aHRs) with 95% confidence intervals (CI). All statistical analyses were performed using IBM SPSS (Statistical Package for the Social Sciences) Statistics for Windows, version 27.0 (International Business Machines Corporation, Armonk, NY, USA). The median follow-up duration, PFS, and OS were calculated using the reverse Kaplan–Meier method and reported in months (mean ± standard error [SE]). A significance level of 0.05 was set for all statistical tests. To reduce selection and immortal-time bias, landmark analyses were performed at 2 and 3 months (≈8 and 12 weeks). Patients who were event-free and under follow-up at the landmark time were included, and the time origin was reset to the landmark. Kaplan–Meier curves and two-group log-rank tests were applied. Twelve- and 24-month survival probabilities were reported with 95% confidence intervals using Greenwood’s method. SUVmax-IPI stratification was performed according to the threshold of 241.9. Comparative analyses were performed to benchmark the prognostic performance of SUVmax-IPI against established systemic inflammatory indices (LIPI, NLR, PLR, SII) and composite models integrating SUVmax-IPI with each index. Performance metrics included time-dependent AUC, Harrell’s concordance index (C-index), ΔC-index, and reclassification measures. Clinical utility was assessed using decision curve analysis (DCA) at 12 and 24 months. The C-index and DCA were computed in R, version 4.3.2 (R Foundation for Statistical Computing, Vienna, Austria). Cox proportional hazards models were fitted with a limited number of covariates in accordance with the events-per-variable (EPV ≈ 10) principle. The proportional hazards assumption was evaluated using Schoenfeld residuals, while multicollinearity was assessed with variance inflation factors (see Supplementary Tables S3–S5). In addition, sensitivity analyses were performed using ridge-penalized Cox regression to reduce potential overfitting. Finally, to directly address the comparative prognostic value of the SUVmax-IPI score, cox proportional hazards regression models and likelihood ratio tests were used to compare SUVmax-IPI against its individual components, SUVmax and IPI.

## 3. Results

### 3.1. Patient Characteristics

The analysis included 187 individuals with a median age of 66 years (interquartile range [IQR], 60–70 years). The cohort comprised 159 male (85%) and 28 female (15%) patients. Histological distribution was adenocarcinoma (49.2%), squamous cell carcinoma (48.1%), and other subtypes (2.7%). PD-L1 expression status was positive (26.7%), negative (44.9%), or undetermined (28.3%), with median expression of 17.5% (IQR, 1–50%). At initial diagnosis, 59.1% of cases presented with stage IV disease. Common sites of metastasis included pulmonary (55.1%), osseous (41.7%), distant nodal (42.8%), cerebral (20.3%), hepatic (10.2%), and other locations (29.4%).

Nivolumab was initiated as second-line therapy in 73.8% of the study cohort. The best overall responses to nivolumab were as follows: a partial response (PR) in 51.1% of participants, a complete response (CR) in 13.0%, stable disease (SD) in 19.6%, while 16.3% had progressive disease (PD). Immune-related adverse events (irAEs) occurred in 21.9% of patients, with the most common being thyroiditis (34.1%), cutaneous adverse reactions (24.4%), and pneumonitis (22.0%).

PET imaging analysis yielded a median SUVmax of 11.6 (IQR, 7.5–16.2). ROC curve analysis established 241.9 as the optimal SUVmax-IPI threshold (area under the curve, 0.57). The median SUVmax-IPI score across the cohort was 116.8 (IQR, 32.7–446.7). Using this validated cutoff, 65.2% of participants showed SUVmax-IPI scores at or below 241.9, compared with 34.8% above this threshold. Significant between-group differences were observed for sex (*p* = 0.014), histological subtype (*p* = 0.001), and median SUVmax values (*p* < 0.001), as presented in Table 1.

### 3.2. Univariate and Multivariate Analysis of Progression-Free Survival and Overall Survival

Cox regression analyses were conducted to assess potential prognostic factors for PFS and overall survival OS. Univariate analysis demonstrated that former smoker status (hazard ratio [HR], 1.63; *p* = 0.028), greater number of prior treatment lines (HR, 1.29; *p* < 0.001), progressive disease (HR, 12.85; 95% CI, 7.71–21.42; *p* < 0.001 vs. complete response), and stable disease (HR, 4.36; 95% CI, 2.62–7.26; *p* < 0.001 vs. complete response) were significantly associated with disease progression. Notably, both unknown PD-L1 status (HR, 0.56; 95% CI, 0.34–0.91; *p* = 0.018) and immune-related adverse events (HR, 0.45; 95% CI, 0.26–0.78; *p* = 0.004) were identified as protective factors against disease progression.

Multivariate analysis confirmed de novo metastasis (HR, 1.71; 95% CI, 1.09–2.68; *p* = 0.019), best response to nivolumab (partial response: HR, 2.23; 95% CI, 1.01–4.92; *p* = 0.047; stable disease: HR, 4.42; 95% CI, 2.12–9.21; *p* = 0.001; progressive disease: HR, 11.02; 95% CI, 5.32–22.81; *p* < 0.001), and immune-related adverse events (HR, 0.45; 95% CI, 0.26–0.79; *p* = 0.006) as independent predictors of PFS (Table 2).

For OS, univariate analysis identified ex-smoker status (HR, 1.76; 95% CI, 1.05–2.97; *p* = 0.033), the presence of brain metastasis (HR, 1.66; 95% CI, 1.00–2.76; *p* = 0.049), and disease progression (HR, 24.42; 95% CI, 7.71–77.39; *p* < 0.001) as significant risk factors. An SUVmax-IPI greater than 241.9 was associated with approximately 2-fold increased mortality risk (HR, 1.96; 95% CI, 1.27–3.02; *p* = 0.002).

The multivariate model confirmed PD-L1 positive status (HR, 2.03; 95% CI, 1.08–3.83; *p* = 0.028), unknown PD-L1 status (HR, 2.38; 95% CI, 1.11–5.13; *p* = 0.027), and disease progression (HR, 28.77; 95% CI, 8.26–100.21; *p* < 0.001) as independent risk factors. SUVmax-IPI greater than 241.9 maintained prognostic significance (HR, 2.50; 95% CI, 1.39–4.49; *p* = 0.002), with patients demonstrating significantly shorter median OS (15 months vs. 35 months; *p* = 0.002). The complete results of the univariate and multivariate analyses for OS are presented in Table 3. Model diagnostics confirmed that the proportional hazards assumption was not violated, and no significant multicollinearity was observed. Sensitivity analyses with ridge-penalized Cox regression produced results consistent with the primary models, supporting the robustness of our findings. These additional analyses are presented in the Supplementary Files.

### 3.3. Survival Outcomes

**Figure 1:**
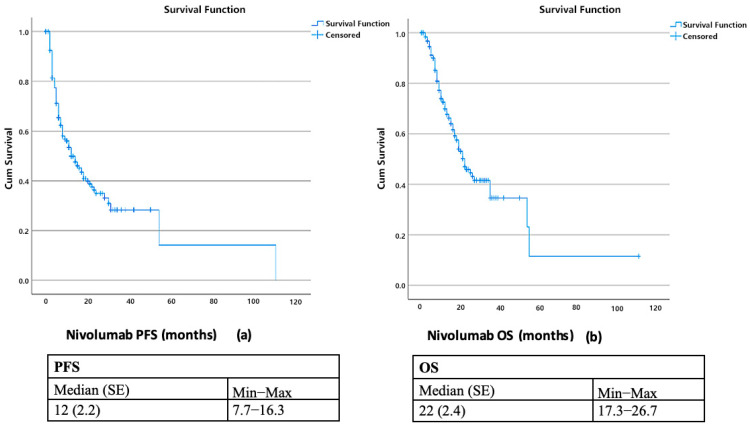
Kaplan–Meier Analysis of Progression-Free Survival (**a**) and Overall Survival (**b**) with Nivolumab Treatment.

**Figure 2:**
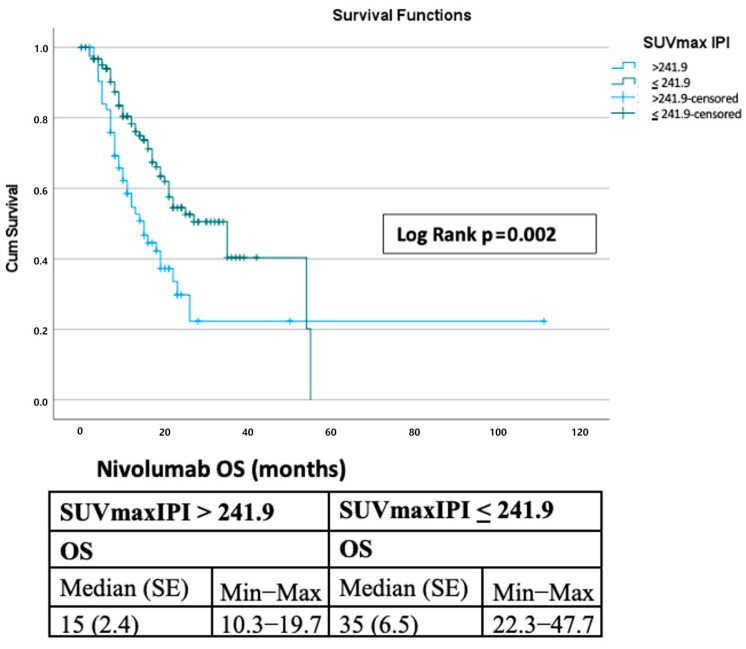
Kaplan–Meier Survival Analysis of Overall Survival in Nivolumab-Treated Patients: SUVmax IPI > 241.9 versus ≤241.9.

**Figure 3:**
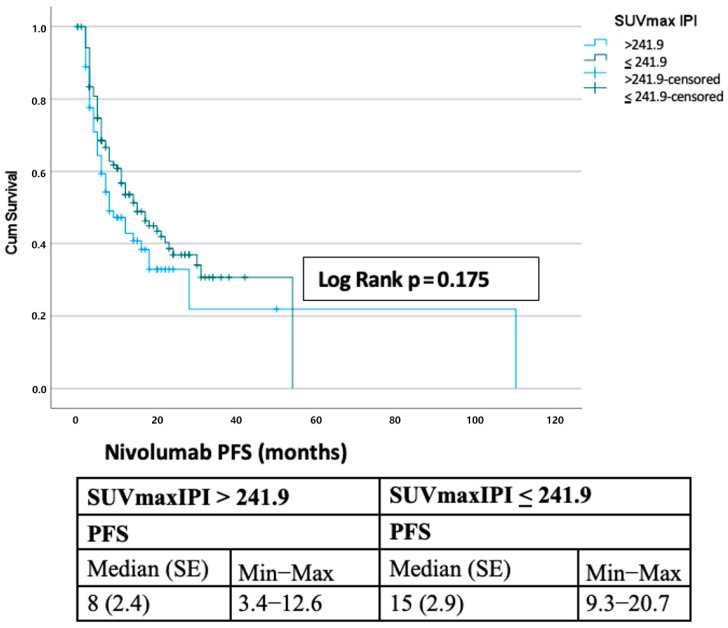
Kaplan–Meier Analysis of Progression-free Survival in Nivolumab-Treated Patients: SUVmax-IP1 > 241.9 versus ≤241.9.

The median progression-free survival was 12 months, with 1-, 2-, 3-, and 5-year PFS rates of 49.9%, 35.0%, 28.4%, and 14.2%, respectively. Median overall survival was 22 months (SE, 2.4; 95% CI, 17.3–26.7), with corresponding survival rates of 69.9%, 45.9%, 34.6%, and 11.5% (Figure 1). Stratification by the SUVmax-IPI threshold (241.9) revealed significant prognostic differences. Patients with SUVmax-IPI values > 241.9 had significantly shorter OS than those with values ≤ 241.9 (median OS: 15 vs. 35 months; log-rank *p* = 0.002). This difference was observed at both 1-year (78.3% vs. 58.6%) and 2-year (54.5% vs. 29.8%) timepoints, with progressively diverging survival curves (Figure 2). By contrast, PFS did not differ significantly between groups; median PFS was 8.0 vs. 15.0 months for SUVmax-IPI > 241.9 vs. ≤ 241.9, with a log-rank *p* = 0.175 (Figure 3).

Landmark analyses confirmed the prognostic impact of SUVmax-IPI. At the 2-month landmark (N = 181), patients in the low SUVmax-IPI group had a median OS of 33 months compared to 13 months in the high group, with 1- and 2-year OS rates of 76.8% versus 50.8% and 54.0% versus 22.4%, respectively (log-rank *p* = 0.00050). Unadjusted Cox hazard ratios (High vs Low SUVmax-IPI group) detailed in Supplementary Table S6. At the 3-month landmark (N = 177), median OS was 30 versus 12 months, with 1- and 2-year rates of 74.2% versus 48.4% and 52.0% versus 20.8% (log-rank *p* = 0.00092). For PFS, the median values at the 2-month landmark (N = 167) were 16 versus 10 months (1-year PFS: 54.4% vs. 45.9%; 2-year: 39.2% vs. 37.0%; log-rank *p* = 0.00868). At the 3-month landmark (N = 148), the median PFS was 19 versus 13 months, with no significant difference between groups (1-year: 58.7% vs. 52.6%; 2-year: 44.3% vs. 42.4%; log-rank *p* = 0.342). Kaplan–Meier curves for OS and PFS at 8- and 12-week landmark analyses are presented in Supplementary Figures S1 and S2, respectively.

### 3.4. Time-Dependent ROC/C-Index and Calibration

Time-dependent discrimination analysis showed modest performance of SUVmax-IPI. For OS, time dependent AUC values were 0.368 at 12 months and 0.337 at 24 months (see Supplementary Table S7). For PFS, corresponding time dependent AUC values were 0.443 and 0.395, respectively. The Harrell’s C-index for OS was 0.587, with bootstrap correction yielding 0.583 (95% CI, 0.523–0.651) (see Supplementary Table S8). For PFS, the corrected C-index was 0.525 (95% CI, 0.472–0.599). Calibration curves demonstrated reasonable concordance between predicted and observed OS at 12 months. Detailed calibration plots and restricted cubic spline analyses are provided in Supplementary Figures S3–S6.

### 3.5. Comparison with Other Prognostic Scores

SUVmax-IPI was benchmarked against LIPI, NLR, PLR, and SII, as well as composite models integrating these indices. Overall, SUVmax-IPI showed comparable or superior prognostic performance, with time-dependent AUC and C-index consistently supporting its discriminatory value. Reclassification metrics indicated modest incremental improvements when combined with other indices. Detailed comparative results are presented in Supplementary Tables S9 and S10. Decision curve analysis at 12 and 24 months further demonstrated a higher net clinical benefit compared with single inflammatory markers (see Supplementary Figures S7 and S8).

### 3.6. Comparative Performance of SUVmax-IPI Against IPI and SUVmax Alone

A comparative analysis was conducted to evaluate the prognostic performance of the SUVmax-IPI relative to its individual components. In univariate Cox regression analysis, SUVmax alone was not significantly associated with overall survival (HR = 1.06, *p* = 0.805). In contrast, the IPI demonstrated a significant association with survival outcomes (HR = 1.66; 95% CI, 1.06–2.60; *p* = 0.028). The composite SUVmax-IPI showed significant prognostic value in both univariate (HR = 1.96; 95% CI, 1.27–3.02; *p* = 0.002) and multivariate analyses (HR = 1.97; 95% CI, 1.06–3.66; *p* = 0.032). Model comparisons using Likelihood Ratio tests indicated that the SUVmax-IPI model provided a significantly better fit than the SUVmax-alone model (Δ−2LL = 8.91, *p* = 0.003). When compared to the IPI-alone model, the SUVmax-IPI model showed a marginally significant improvement in model fit (Δ−2LL = 3.91, *p* = 0.048). The SUVmax-IPI score demonstrated statistically superior model fit compared to SUVmax alone, as evidenced by its lower −2LL value and significant likelihood ratio test results, while also maintaining independent prognostic value in the multivariate analysis.

## 4. Discussion

In this multicenter study, the results showed that a high SUVmax-IPI score is significantly associated with shorter overall survival in patients with NSCLC treated with nivolumab, supporting the clinical utility of this composite biomarker for risk stratification. To the best of our knowledge, this is the first study to combine FDG-PET-derived SUVmax with the IPI to develop the novel SUVmax-IPI for outcome prediction in advanced NSCLC treated with nivolumab.

The metabolic component SUVmax reflects fundamental shifts in tumor biology where elevated glycolytic activity promotes immune evasion through lactate-mediated suppression of cytotoxic T-cells [7]. Prior research has suggested that SUVmax of primary tumors may serve as a prognostic indicator in patients with NSCLC [12,13]. However, conflicting evidence exists, as demonstrated by Machtay et al. in a large prospective study of 226 patients with advanced NSCLC, which found no significant correlation between pretreatment SUVmax and survival outcomes [14]. Similarly, Guo et al. reported in their investigation of SUVmax and NLR in locally advanced NSCLC that SUVmax was not an independent risk factor [15]. Consistent with these findings, our study also found no significant association between SUVmax values and either PFS or OS. These collective discrepancies may be attributable to heterogeneity in patient demographics and clinical features across studies.

In light of these discrepancies, researchers have increasingly focused on incorporating inflammatory markers to enhance prognostic accuracy, particularly for immunotherapy. The LIPI, which combines dNLR and lactate dehydrogenase (LDH) levels, has emerged as a validated tool for this purpose. Mezquita et al. reported that patients who had poor LIPI scores showed significantly shorter PFS and OS after receiving immune checkpoint inhibitors [9]. Subsequent studies by Liu et al. and Chen et al. independently demonstrated that elevated baseline levels of NLR, dNLR, SII, and PLR were all significantly associated with worse outcomes in patients with NSCLC treated with nivolumab, highlighting the impact of systemic inflammation on response to immune checkpoint inhibition [10,16]. A comprehensive systematic review by Rebuzzi et al. examined 22 distinct prognostic scores incorporating inflammatory markers in patients with NSCLC receiving immune checkpoint inhibitors [17]. Their analysis not only confirmed the clinical utility of LIPI but also highlighted the consistent advantage of multivariable composite models over single biomarkers for predicting immunotherapy responses. Similarly, Rizzo et al. demonstrated that NLR and LDH retained prognostic significance in patients with advanced NSCLC undergoing immunotherapy, further validating the role of inflammatory indices in risk stratification [18].

Beyond hematologic indices, albumin-related inflammatory markers have gained recognition as valuable prognostic tools. A comprehensive review of nutritional-inflammatory indices, including the prognostic nutritional index (PNI), C-reactive protein-to-albumin ratio (CAR), and albumin-to-globulin ratio, highlights their dual role in reflecting both nutritional status and systemic inflammation [19]. This systematic evaluation of clinical evidence consistently identified hypoalbuminemia and elevated CRP-based scores as strong predictors of adverse outcomes [19]. The review’s findings are further supported by subsequent validation of the IPI, which incorporates albumin, NLR, and CRP, demonstrating its prognostic utility across different stages of NSCLC [11]. Moreover, recent investigations have advanced the development of composite biomarkers combining metabolic and inflammatory parameters. A study of patients with stage IIIB to IV NSCLC who received chemotherapy demonstrated that a composite score combining SUVmax and lymphocyte-to-monocyte ratio independently predicted both progression-free and overall survival [20]. This work highlighted the synergistic prognostic value of assessing both tumor metabolic activity and host inflammatory status [20]. Building upon this foundation, we adapted this innovative approach to the immunotherapy setting by integrating SUVmax with the more comprehensive IPI. Our model expands beyond previous methodologies by incorporating albumin and CRP measurements, thereby capturing a wider spectrum of systemic inflammation and nutritional status parameters. Similarly, Guo et al. developed the SUVmax-NLR (SNG) score for patients with locally advanced NSCLC, demonstrating better prognostic performance than individual biomarkers [15]. In contrast, our study specifically evaluated outcomes in patients with advanced NSCLC who received nivolumab, offering novel insights into prognostication specific to immune checkpoint inhibitors.

Complementing these findings, Ke et al. demonstrated that combining SUVmax with elevated LDH levels significantly improved prognostic stratification in patients with non-small cell and small cell lung cancer receiving chemoimmunotherapy [21]. Their analysis revealed that this composite biomarker showed significant association with overall. survival even when neither parameter was independently predictive, highlighting the potential of metabolic-inflammatory combinations in immune checkpoint inhibitor-based therapies [20]. In contrast to Ke et al.’s study, our investigation differs in three critical aspects: First, while their cohort included patients with both non-small cell and small cell lung cancer, we focused exclusively on metastatic NSCLC. Second, we evaluated patients receiving nivolumab monotherapy rather than combination treatment regimens. Third, and most importantly, we utilized the IPI incorporating albumin, CRP, and NLR instead of LDH as the inflammatory component. This design provides a more immunologically focused evaluation by eliminating the confounding effects of chemotherapy, thereby offering clearer insights into prognostic factors specific to PD-1 inhibition.

Regarding the finding that PD-L1 positive and unknown categories were associated with higher mortality, potential explanations include the retrospective study design, missing PD-L1 results in patients with poorer status, and the restriction of nivolumab use to later treatment lines in our country. Notably, after adjustment for confounders such as treatment line and brain metastasis, this association persisted and should therefore be interpreted with caution and validated in prospective studies.

The prognostic value of combining SUVmax with IPI was evaluated through comparative analysis. The results demonstrate that while SUVmax alone lacked prognostic significance, the composite SUVmax-IPI score provided significant prognostic value. Model comparisons revealed that SUVmax-IPI offered superior performance to SUVmax alone and showed marginally better fit than IPI alone. These findings suggest that baseline tumor metabolic activity (captured by SUVmax) may provide complementary information beyond inflammation-based markers.

Our study has certain limitations that should be considered when interpreting the findings. First, the inclusion criterion of having completed at least three months of nivolumab treatment means that our results are most applicable to this specific patient population, and may not be generalizable to those who discontinue treatment earlier. Second, the cut-off value for the SUVmax-IPI was identified using Youden’s index within this cohort. While this is a standard approach for exploratory biomarker development, it inherently carries a risk of overfitting, and thus, this specific threshold requires validation in an independent cohort before any clinical application can be considered.

In summary, SUVmax-IPI score, which integrates metabolic and inflammatory parameters, may represent a practical and non-invasive approach to prognostication in patients with metastatic NSCLC treated with nivolumab. By combining FDG-PET–derived SUVmax with routine laboratory indices, this model has the potential to improve risk stratification and support clinical decision-making. However, given the retrospective design these findings should be interpreted as exploratory and hypothesis-generating. Prospective studies with larger, independent cohorts are warranted to validate the clinical applicability of this index.

## 5. Conclusions

The SUVmax-IPI score, which combines metabolic and inflammatory biomarkers, emerged as an independent predictor of overall survival but not of PFS in patients with metastatic NSCLC treated with nivolumab. By integrating FDG-PET data with routine laboratory parameters, this score may provide incremental value for prognostication. However, the retrospective design, modest discrimination metrics, cohort-specific cut-off, and the inclusion criterion requiring a minimum of three months of therapy necessitate that these findings be interpreted as exploratory and hypothesis-generating. External validation is required before clinical application.

## Figures and Tables

**Table 1:** Demographic and Clinical Characteristics of Patients.

				SUVmax IPI				
n = 187	Total			>241.9		≤241.9		
		n	%	n	%	n	%	*p*
Gender	Male	159	85.0	61	93.8	98	80.3	0.014
	Female	28	15.0	4	6.2	24	19.7	
Age at Diagnosis	Median (IQR)	66	(60–70)	66 (60–70)		65.5 (58.75–70.25)		0.764
Smoking Status	Smoker	63	33.7	24	36.9	39	32.0	0.495
	Ex-smoker	124	66.3	41	63.1	83	68.0	
Histological Type	Adenocarcinoma	92	49.2	21	32.3	71	58.2	0.001
	Squamous cell	90	48.1	43	66.2	47	38.5	
	Other	5	2.7	1	1.5	4	3.3	
Mutational Status	Unknown	11	5.9	4	6.2	7	5.7	0.938
	Positive	11	5.9	3	4.6	8	6.6	
	Negative	165	88.2	58	89.2	107	87.7	
PD-L1 Status	Unknown	53	28.3	20	30.8	33	27.0	0.863
	Positive	50	26.7	17	26.2	33	27.0	
	Negative	84	44.9	28	43.1	56	45.9	
Stage at Initial Diagnosis	1	4	2.2	1	1.5	3	2.5	0.564
	2	11	5.9	6	9.2	5	4.1	
	3	61	32.8	20	30.8	41	33.9	
	4	110	59.1	38	58.5	72	59.5	
Metastatic Pattern	de novo	116	62.0	41	63.1	75	61.5	0.830
	Recurrence	71	38.0	24	36.9	47	38.5	
SUVmax	Median (IQR)	11.6	(7.5–16.2)	15.1 (11.2–20.7)		9.65 (7.5–13.5)		˂0.001
Site of Metastasis	Lung	103	55.1	36	55.4	67	54.9	0.951
	Liver	19	10.2	9	13.8	10	8.2	0.223
	Brain	38	20.3	11	16.9	27	22.1	0.399
	Bone	78	41.7	31	47.7	47	38.5	0.226
	Distant lymph node	80	42.8	25	38.5	55	45.1	0.384
	Other	55	29.4	55	29.4	19	29.2	0.968
Prior Therapy Before Nivolumab	Pemetrexed combination	27	14.6	6	9.4	21	17.4	0.300
	Taxane combination	84	45.4	33	51.6	51	42.1	
	Gemcitabine combination	67	36.2	24	37.5	43	35.5	
	Other	7	3.8	1	1.6	6	5.0	
Treatment Line of Nivolumab	1	3	1.6	1	1.5	2	1.6	0.0717
	2	138	73.8	49	75.4	89	73.0	
	3	33	17.6	13	20.0	20	16.4	
	4	9	4.8	1	1.5	8	6.6	
	5	3	1.6	1	1.5	2	1.6	
	6	1	0.5	0	0.0	1	0.8	
Total number of lines of treatment	Median (IQR)	3	(2–3)	2 (2–3)		3 (2–3)		0.214
Disease Progression	Yes	108	57.8	41	63.1	67	54.9	0.282
	No	79	42.2	24	36.9	55	45.1	
Reason for Nivolumab Cessation	Progression	105	93.8	40	93.0	65	94.2	0.536
	Hyperprogression	1	0.9	0	0.0	1	1.4	
	Adverse effect	1	0.9	0	0.0	1	1.4	
	Follow-up after CR	1	0.9	0	0.0	1	1.4	
	Other	4	3.6	3	7.0	1	1.4	
Best Response to Nivolumab Treatment	CR	27	14.4	11	16.4	16	13.3	0.465
	PR	94	50.2	29	43.2	65	54.2	
	SD	36	19.2	13	19.4	23	19.2	
	PD	30	16.0	14	20.8	16	13.3	
Nivolumab Related Adverse Events	No	146	78.07	58	85.3	88	73.9	0.107
	Yes	41	21.92	10	14.7	31	26.1	
Grade of Nivolumab Related Adverse Events	Grade 1	18	43.9	8	80.0	10	32.3	0.050
	Grade 2	19	46.3	2	20.0	17	54.8	
	Grade 3	1	2.4	0	0.0	1	3.2	
	Grade 4	3	7.3	0	0.0	3	9.7	
Types of Nivolumab Related Adverse Events	Colitis	2	4.9	0	0.0	2	6.5	0.493
	Hepatitis	1	2.4	1	10.0	0	0.0	
	Thyroiditis	15	34.1	3	30.0	11	35.5	
	Skin-related	10	24.4	2	20.0	8	25.8	
	Pneumonitis	10	22.0	3	30.0	6	19.4	
	Myocarditis	1	2.4	1	10.0	0	0.0	
	Other	2	4.9	0	0.0	2	6.5	
	Arthritis	1	2.4	0	0.0	1	3.2	
Treatments for Nivolumab Related Adverse Events	Steroid	11	26.8	3	30.0	8	25.8	1.000
	Steroid and other	1	2.4	0	0.0	1	3.2	
	Topical	9	22.0	2	20.0	7	22.6	
	Other	20	48.8	5	50.0	15	48.4	
Rechallenge with Nivolumab After Adverse Events	No	3	7.3	0	0.0	3	10.3	1.000
	Yes	38	92.7	12	100	26	89.7	
Death	No	102	54.5	27	41.5	75	61.5	0.009
	Yes	85	45.5	38	58.5	47	38.5	
Follow-up period (months)	Median (IQR)	28 (18–42)		25 (16–38.5)		31.5 (19.75–42.25)		0.049

Data are presented as number (n) of patients and percentage (%). Abbreviations: SUVmax, maximum standardized uptake value; IPI, inflammatory prognostic index; IQR, interquartile range; PD-L1, programmed death-ligand 1; CR, complete response; PR, partial response; SD, stable disease; PD, progressive disease.

**Table 2:** Cox regression analysis of progression-free survival.

	Univariate		Multivariate	
	*p*	HR (95% Cl Min–Max)	*p*	HR (95% Cl Min–Max)
Gender Female	0.227	1.360 (0.826–2.238)		
Age at Diagnosis	0.061	1.024 (0.999–1.049)	0.411	1.011 (0.985–1.038)
Smoking Status Ex-Smoker	0.028	1.634 (1.054–2.533)	0.093	1.542 (0.931–2.555)
Histological Type (Ref: Adenocarcinoma)	0.737			
Squamous cell	0.437	1.167 (0.791–1.721)		
Other	0.955	1.034 (0.321–3.331)		
Mutation Status (Ref: Negative)	0.286			
Unknown	0.296	1.507 (0.698–3.252)		
Positive	0.256	0.512 (0.161–1.624)		
PD-L1 Status (Ref: Negative)	0.061		0.244	
Unknown	0.018	0.556 (0.341–0.905)	0.095	0.624 (0.359–1.085)
Positive	0.556	0.872 (0.552–1.377)	0.665	0.893 (0.534–1.493)
PD-L1 Positivity Rate	0.241	0.993 (0.982–1.005)		
Stage at Initial Diagnosis (Ref: 4)	0.454			
Stage 1	0.178	0.257 (0.035–1.854)		
Stage 2	0.534	1.264 (0.604–2.644)		
Stage 3	0.591	0.891 (0.585–1.357)		
Metastatic Pattern Recurrence	0.213	1.288 (0.865–1.916)	0.019	
SUVmax	0.201	0.983 (0.957–1.009)	0.623	0.990 (0.951–1.031)
Lung Metastasis	0.472	1.153 (0.783–1.698)		
Liver Metastasis	0.805	1.083 (0.576–2.034)		
Brain Metastasis	0.081	1.503 (0.951–2.375)	0.325	1.322 (0.759–2.303)
Bone Metastasis	0.146	1.330 (0.905–1.953)	0.389	1.200 (0.792–1.817)
Distant Lymph Node Metastasis	0.627	1.100 (0.749–1.617)		
Other Metastasis	0.537	0.875 (0.573–1.337)		
Prior Therapy Before Nivolumab (Ref: pemetrexed combination)	0.450			
taxan combination	0.113	1.669 (0.887–3.141)		
gemcitabine combination	0.220	1.502 (0.784–2.877)		
other	0.695	1.259 (0.398–3.987)		
Treatment Line of Nivolumab	0.459	0.901 (0.683–1.188)		
Total number of lines of Treatment	˂0.001	1.285 (1.123–1.470)	0.133	1.144 (0.960–1.363)
Best Response to Nivolumab Treatment (Ref: CR)	˂0.001		˂ 0.001	
PR	0.112	1.848 (0.866–3.941)	0.047	2.231 (1.011–4.291)
SD	˂0.001	4.363 (1.966–9.683)	0.001	4.416 (1.905–10.237)
PD	˂0.001	12.847 (5.628–29.327)	˂0.001	11.018 (4.383–27.696)
Nivolumab- Related Adverse Events	0.004	0.451 (0.261–0.781)	˂0.001	0.449 (0.253–0.798)
SUVmaxIPI > 241.9	0.191	1.303 (0.876–1.938)	0.086	1.532 (0.942–2.492)

Abbreviations: HR, hazard ratio; CI, confidence interval; Ref, reference; PD-L1, programmed death-ligand 1; SUVmax, maximum standardized uptake value; PR, partial response; SD, stable disease; PD, progressive disease; CR, complete response; IPI, inflammatory prognostic index.

**Table 3:** Cox regression analysis of overall survival.

	Univariate		Multivariate	
	*p*	HR (95%CI Min–Max)	*p*	HR (95%CI Min–Max)
Gender Female	0.325	1.325 (0.757–2.321)		
Age at Diagnosis	0.080	1.025 (0.997–1.053)	0.934	0.999 (0.968–1.030)
Smoking Status Ex-Smoker	0.033	1.763 (1.046–2.971)	1.030	1.866 (0.877–3.791)
Histological Type (Ref: Adenocarcinoma)	0.266			
Squamous cell	0.127	1.409 (0.907–2.190)		
Other	0.773	0.810 (0.195–3.376)		
Mutation Status (Ref: Negative)	0.436			
Unknown	0.881	0.926 (0.339–2.532)		
Positive	0.199	0.274 (0.038–1.977)		
PD-L1 Status (Ref: Negative)	0.223		0.027	
Unknown	0.552	0.851 (0.501–1.446)	0.027	2.380 (1.105–5.126)
Positive	0.202	1.388 (0.839–2.299)	0.028	2.034 (1.081–3.828)
PD-L1 Positivity Rate	0.527	0.996 (0.984–1.008)		
Stage at Initial Diagnosis (Ref: 4)	0.718			
stage 1	0.958	0.000		
stage 2	0.733	1.159 (0.496–2.705)		
stage 3	0.306	0.780 (0.484–1.256)		
Metastatic Pattern (Ref: de novo) Recurrence	0.093	0.677 (0.430–1.067)	0.027	2.010 (1.083–3.732)
SUVmax	0.914	0.998 (0.970–1.027)		
Lung Metastasis	0.359	1.227 (0.793–1.900)		
Liver Metastasis	0.883	1.054 (0.522–2.127)		
Brain Metastasis	0.049	1.663 (1.002–2.762)	0.350	1.393 (0.695–2.795)
Bone Metastasis	0.312	1.250 (0.811–1.925)		
Distant Lymph Node Metastasis	0.761	0.935 (0.604–1.445)		
Other Metastasis	0.223	0.735 (0.448–1.206)	0.428	0.790 (0.441–1.414)
Prior Therapy Before Nivolumab (Ref: pemetrexed combination)	0.154		0.842	
taxan combination	0.051	2.053 (0.998–4.223)	0.741	1.157 (0.488–2.739)
gemcitabine combination	0.252	1.556 (0.730–3.319)	0.727	1.175 (0.475–2.902)
other	0.944	0.954 (0.252–3.613)	0.588	0.633 (0.121–3.309)
Treatment Line of Nivolumab	0.882	0.979 (0.738–1.298)		
Total number of lines of treatment	0.654	1.036 (0.887–1.211)		
Progression	˂0.001	24.422 (7.706–77.394)	˂0.001	28.774 (8.262–100.205)
Best Response to Nivolumab Treatment (Ref: CR)	0.000		0.062	
PR	0.279	1.500 (0.719–3.129)	0.369	0.661 (0.268–1.630)
SD	0.029	2.421 (1.094–5.360)	0.589	0.760 (0.280–2.060)
PD	˂0.001	4.199 (1.898–9.289)	0.284	1.703 (0.644–4.506)
Nivolumab- Related Adverse Events	0.019	0.480 (0.260–0.887)	0.819	1.086 (0.536–2.198)
SUVmaxIPI > 241.9	0.002	1.961 (1.273–3.022)	0.002	2.499 (1.392–4.486)

Abbreviations: HR, hazard ratio; CI, confidence interval; Ref, reference; PD-L1, programmed death-ligand 1; SUVmax, maximum standardized uptake value; PR, partial response; SD, stable disease; PD, progressive disease; CR, complete response; IPI, inflammatory prognostic index.

## Data Availability

Data will be available from the corresponding author upon reasonable request.

## Supplementary Materials

The following supporting information can be downloaded at: https://www.mdpi.com/article/10.3390/curroncol32100566/s1, Figure S1: Kaplan–Meier curves for OS at 8- and 12-week landmark analyses; Figure S2: Kaplan–Meier curves for PFS at 8- and 12-week landmark analyses; Figure S3: Time-dependent calibration plots (12 and 24 months) for OS; Figure S4: Time-dependent calibration plots (12 and 24 months) for PFS; Figure S5: Restricted cubic spline (RCS) effects of SUVmax-IPI as a continuous predictor of OS; Figure S6: Restricted cubic spline (RCS) effects of SUVmax-IPI as a continuous predictor of PFS; Figure S7: Decision curve analysis (DCA) for OS at 12 and 24 months; Figure S8: Decision curve analysis (DCA) for PFS at 12 and 24 months; Table S1: Variable dictionary and formulas; Table S2: Raw input values and calculated SUVmax-IPI scores for each patient; Table S3: Cox coefficients (per 1 SD), HR and 95% CI; Table S4: Collinearity (VIF); Table S5: Proportional Hazards (approximate test); Supplementary Table S6: Unadjusted Cox Hazard Ratios (High vs Low SUVmax-IPI group); Supplementary Table S7: Time-dependent AUC at 12/24 months and C-index; Supplementary Table S8: Bootstrap-corrected C-index and 95% CI; Supplementary Table S9: Score comparison for OS: SUVmax-IPI vs LIPI, NLR, PLR, SII and composite models (IPI+LIPI, IPI+NLR, IPI+PLR, IPI+SII); Supplementary Table S10: Score comparison for PFS: SUVmax-IPI vs LIPI, NLR, PLR, SII and composite models (IPI+LIPI, IPI+NLR, IPI+PLR, IPI+SII).

## Abbreviations

The following abbreviations are used in this manuscript:

SUVmax	maximum standardized uptake value
IPI	inflammatory prognostic index
FDG-PET	^18^F-fluorodeoxyglucose positron emission tomography
CRP	C-reactive protein
NLR	neutrophil-to-lymphocyte ratio
ROC	receiver operating characteristic
IQR	interquartile range
CI	confidence interval
SE	standard error
aHR	adjusted hazard ratio
SPSS	Statistical Package for the Social Sciences
C-index	concordance index
DCA	decision curve analysis
CR	complete response
PR	partial response
SD	stable disease
PD	progressive disease
irAEs	immune-related adverse events
HR	hazard ratio
LIPI	Lung Immune Prognostic Index
dNLR	derived neutrophil-to-lymphocyte ratio
SII	systemic immune-inflammation index
PLR	platelet-to-lymphocyte ratio
