# Supplementary material for: SUVmax-IPI as a New Prognostic Index in Metastatic Non-Small Cell Lung Cancer Patients Receiving Nivolumab
Source: Curr Oncol. 2025 Oct 9;32(10):566. doi: 10.3390/curroncol32100566 (PMC12564384; doi:10.3390/curroncol32100566)
Supplement: Supplementary file 1 [file curroncol-32-00566-s001.zip › curroncol-3799530-updated Supplementary File.pdf]

Supplementary Report

This report compiles (1) Variable dictionary and formulas, (2) Raw input values -Final SUVmax-IPI values, (3) Model Complexity and Event/Parameter Balance (OS) (4) Landmark analyses at 8 and 12 weeks (OS & PFS) with Kaplan Meir curves and Cox Hazard Ratios (HRs), (5) Time-dependent ROC/C-index with calibration and bootstrap, (6) Restricted cubic spline (RCS)effect plots for SUVmax-IPI as a continuous predictor, (7) Score comparison: SUVmax-IPI vs LIPI, NLR, PLR, SII and composite models, (8) Decision curve analysis (DCA) at 12 and 24 months,

1) Variable dictionary and formulas

Supplementary Table S1. Variable dictionary and formulas

Score / Variable	Definition / Formula
NLR	NLR = Neutrophil / Lymphocyte
SUVmax-IPI	SUVmax-IPI = SUVmax × (CRP × NLR / Albumin)

Example calculation (one patient):

Patient	SUVmax	CRP	NLR	Albumin	SUVmax-IPI (calc)
HA	1.8	2.98	3.101	4.16	3.998

2) Raw input values and calculated SUVmax-IPI scores for each patient.

Supplementary Table S2. Raw input values and calculated SUVmax-IPI scores for each patient.

Patient_ID	SUVmax (PET)	CRP	Nötrofil	Lenfosit	NLR	Albumin	SUVmax-IPI
0.0	25.5	2.0	4320.0	840.0	5.143	4.0	65.571
1.0	14.3	2.0	5670.0	1780.0	3.185	4.4	20.705
2.0	25.3	40.0	11640.0	2250.0	5.173	3.9	1342.414
3.0	19.9	60.0	4580.0	2510.0	1.825	4.5	484.154
4.0	11.6	6.7	4020.0	820.0	4.902	4.2	90.718
5.0	8.1	11.0	5190.0	1860.0	2.79	4.2	59.195
6.0	11.2	115.0	6370.0	970.0	6.567	3.5	2416.66
7.0	19.9	97.0	7700.0	1790.0	4.302	3.7	2244.196

8.0	27.9	27.0	5190.0	1230.0	4.22	4.2	756.8
9.0	11.8	2.0	3710.0	2100.0	1.767	4.0	10.423
10.0	34.6	2.0	5970.0	2640.0	2.261	4.2	37.259
11.0	19.8	26.0	1610.0	1820.0	0.885	3.9	116.769
12.0	15.1	101.0	7200.0	3460.0	2.081	4.0	793.405
13.0	17.6	18.8	15130.0	1470.0	10.293	3.9	873.228
14.0	28.3	298.0	10800.0	1710.0	6.316	3.1	17181.8
15.0	29.6	46.0	3620.0	3220.0	1.124	3.8	402.827
16.0	25.2	2.0	9040.0	1670.0	5.413	4.2	64.958
17.0	18.9	3.0	5740.0	2890.0	1.986	4.2	26.813
18.0	15.6	3.2	4260.0	2340.0	1.821	4.1	22.166
19.0	17.3	13.0	6950.0	680.0	10.221	4.0	574.653
20.0	31.3	9.9	9930.0	1010.0	9.832	3.9	781.165
21.0	10.1	27.0	3570.0	2330.0	1.532	3.8	109.955
22.0	12.5	8.0	6260.0	1160.0	5.397	0.13	4151.194
23.0	23.5	46.0	3420.0	1460.0	2.342	4.2	602.906
24.0	18.4	4.9	2840.0	1340.0	2.119	3.7	51.645
25.0	10.6	23.0	3520.0	960.0	3.667	3.1	288.366
26.0	16.9	2.0	4560.0	2940.0	1.551	2.8	18.723
27.0	12.9	23.0	8290.0	3280.0	2.527	3.1	241.9
28.0	16.2	8.9	5550.0	1190.0	4.664	3.7	181.739
29.0	20.4	2.0	2480.0	890.0	2.787	4.1	27.729
30.0	11.6	4.0	2940.0	1110.0	2.649	3.7	33.215
31.0	19.2	8.6	3220.0	1080.0	2.981	3.5	140.658
32.0	19.9	2.3	4510.0	2640.0	1.708	3.9	20.049
33.0	16.4	48.0	9930.0	9100.0	1.091	4.0	214.75
34.0	13.5	3.8	3880.0	1940.0	2.0	4.6	22.304
35.0	12.5	22.5	6300.0	8100.0	0.778	3.7	59.122
36.0	7.5	1.9	3870.0	1870.0	2.07	3.8	7.761
37.0	34.1	67.0	7300.0	1490.0	4.899	3.7	3025.269
38.0	22.1	26.0	10000.0	890.0	11.236	4.2	1537.186
39.0	5.3	3.2	3740.0	1310.0	2.855	4.0	12.105
40.0	13.3	7.8	5400.0	1700.0	3.176	3.9	84.494
41.0	26.9	20.8	5170.0	2560.0	2.02	3.8	297.36
42.0	16.7	2.6	4400.0	1610.0	2.733	4.4	26.969
43.0	23.6	3.9	6300.0	3280.0	1.921	4.3	41.113
44.0	8.0	4.0	4820.0	4670.0	1.032	4.7	7.027
45.0	13.7	10.9	3580.0	1780.0	2.011	3.9	77.01
46.0	15.4	3.8	7290.0	2190.0	3.329	4.0	48.7
47.0	18.0	87.0	3610.0	1040.0	3.471	4.7	1156.559
48.0	16.0	18.0	5700.0	2350.0	2.426	3.9	179.116
49.0	21.0	35.9	7220.0	1160.0	6.224	4.1	1144.482
50.0	14.2	20.0	8440.0	1800.0	4.689	4.0	332.911
51.0	15.6	65.0	9440.0	2550.0	3.702	4.3	872.974
52.0	30.0	28.0	5340.0	1630.0	3.276	3.4	809.383
53.0	20.4	53.0	5600.0	1800.0	3.111	3.9	862.496
54.0	15.3	1.4	3290.0	880.0	3.739	4.1	19.532
55.0	15.3	100.0	11000.0	1980.0	5.556	4.0	2125.0

56.0	21.5	9.7	6140.0	1610.0	3.814	4.3	184.963
57.0	25.5	13.0	4950.0	2150.0	2.302	3.6	212.006
58.0	5.5	7.0	5390.0	1080.0	4.991	4.0	48.036
59.0	14.3	47.4	7090.0	3480.0	2.037	3.9	354.093
60.0	20.1	160.0	6710.0	1080.0	6.213	3.7	5400.24
61.0	16.3	99.0	6780.0	3260.0	2.08	3.3	1017.0
62.0	15.8	1.4	2810.0	960.0	2.927	3.7	17.499
63.0	12.6	28.8	8390.0	1390.0	6.036	3.4	644.216
64.0	15.9	63.1	7070.0	1030.0	6.864	3.7	1861.26
65.0	12.8	26.0	5900.0	3550.0	1.662	3.2	172.845
66.0	11.4	9.1	5470.0	2750.0	1.989	4.4	46.897
67.0	17.0	13.9	4650.0	950.0	4.895	3.7	312.602
68.0	9.9	18.8	5470.0	1390.0	3.935	4.2	174.388
69.0	22.9	22.7	7570.0	2500.0	3.028	4.2	374.773
70.0	13.5	94.9	8240.0	940.0	8.766	3.7	3035.272
71.0	11.2	3.2	5050.0	1310.0	3.855	3.8	36.358
72.0	9.9	9.3	3430.0	1160.0	2.957	3.9	69.806
73.0	21.3	24.2	8840.0	1710.0	5.17	3.8	701.241
74.0	25.0	2.15	2520.0	1500.0	1.68	4.3	21.0
75.0	6.0	50.94	4020.0	1780.0	2.258	4.3	160.527
76.0	13.0	15.0	6120.0	1650.0	3.709	4.6	157.233
77.0	11.0	3.27	2200.0	1610.0	1.366	4.0	12.288
78.0	12.0	13.97	7680.0	1660.0	4.627	3.9	198.869
79.0	12.7	2.0	4860.0	920.0	5.283	3.7	36.264
80.0	11.0	3.65	2890.0	1280.0	2.258	4.1	22.11
81.0	9.0	6.07	3040.0	2360.0	1.288	3.4	20.697
82.0	13.0	3.0	2900.0	1270.0	2.283	4.0	22.264
83.0	14.0	104.0	7000.0	2470.0	2.834	3.4	1213.622
84.0	9.0	66.0	6270.0	1540.0	4.071	4.4	549.643
85.0	12.0	26.0	5200.0	2200.0	2.364	3.4	216.898
86.0	3.1	2.3	5570.0	1790.0	3.112	4.2	5.283
87.0	10.3	3.5	4870.0	2430.0	2.004	3.9	18.525
88.0	11.2	46.6	9080.0	1930.0	4.705	4.1	598.892
89.0	8.0	21.39	1700.0	1160.0	1.466	3.8	65.995
90.0	29.0	59.37	5520.0	2230.0	2.475	3.0	1420.62
91.0	10.2	1.9	3610.0	1700.0	2.124	4.7	8.756
92.0	12.0	71.85	8230.0	880.0	9.352	3.7	2179.332
93.0	3.7	26.0	7700.0	3280.0	2.348	4.2	53.77
94.0	9.8	27.6	5140.0	1570.0	3.274	4.5	196.782
95.0	9.2	9.8	6150.0	2410.0	2.552	4.3	53.506
96.0	12.5	24.0	4150.0	2002.0	2.073	4.1	151.678
97.0	8.2	46.0	7130.0	1010.0	7.059	4.2	634.002
98.0	5.39	16.0	5520.0	1260.0	4.381	3.9	96.875
99.0	4.49	3.2	4610.0	1440.0	3.201	4.4	10.454
100.0	21.9	8.6	7030.0	970.0	7.247	3.7	368.913
101.0	8.96	3.35	5320.0	2960.0	1.797	4.2	12.845
102.0	4.06	35.0	6190.0	610.0	10.148	4.1	351.699
103.0	13.55	2.38	5630.0	1880.0	2.995	4.36	22.15

104.0	3.25	23.8	4250.0	1680.0	2.53	4.29	45.612
105.0	2.85	4.54	6760.0	1740.0	3.885	4.45	11.296
106.0	19.93	53.04	7460.0	1020.0	7.314	3.99	1937.656
107.0	8.46	11.81	3150.0	2210.0	1.425	4.35	32.738
108.0	3.99	1.6	4070.0	1670.0	2.437	4.2	3.704
109.0	12.27	23.0	2960.0	330.0	8.97	4.1	617.4
110.0	15.65	13.7	1860.0	1090.0	1.706	4.0	91.466
111.0	12.02	2.58	6370.0	1660.0	3.837	4.55	26.154
112.0	5.17	8.34	6220.0	1310.0	4.748	3.92	52.226
113.0	8.19	9.67	4090.0	1850.0	2.211	4.21	41.589
114.0	6.6	62.46	5780.0	1110.0	5.207	4.23	507.47
115.0	11.57	42.42	5700.0	660.0	8.636	3.33	1272.89
116.0	6.19	4.33	5120.0	1490.0	3.436	4.07	22.629
117.0	8.22	5.3	4300.0	1400.0	3.071	4.06	32.958
118.0	40.31	42.42	7430.0	480.0	15.479	2.97	8911.988
119.0	17.6	8.14	3190.0	540.0	5.907	3.98	212.643
120.0	1.8	2.98	4310.0	1390.0	3.101	4.16	3.998
121.0	6.56	5.25	7170.0	1350.0	5.311	4.74	38.59
122.0	14.01	22.12	3770.0	2340.0	1.611	3.18	157.008
123.0	6.0	6.04	7050.0	1200.0	5.875	4.29	49.629
124.0	13.27	8.27	5520.0	1370.0	4.029	4.0	110.544
125.0	12.4	1.72	3100.0	1830.0	1.694	4.03	8.965
126.0	10.02	18.44	5870.0	730.0	8.041	4.68	317.467
127.0	4.67	0.3	2280.0	1100.0	2.073	4.45	0.653
128.0	5.2	0.76	2930.0	3010.0	0.973	4.2	0.916
129.0	10.84	85.7	4450.0	1470.0	3.027	2.75	1022.634
130.0	9.28	8.57	13270.0	2390.0	5.552	4.06	108.762
131.0	15.02	94.77	11230.0	1310.0	8.573	3.37	3620.924
132.0	10.41	3.36	10210.0	630.0	16.206	4.31	131.522
133.0	5.7	10.7	4150.0	310.0	13.387	4.89	166.969
134.0	19.82	8.5	5560.0	940.0	5.915	4.3	231.74
135.0	11.25	35.8	4910.0	580.0	8.466	4.28	796.609
136.0	11.57	46.98	7660.0	1370.0	5.591	3.15	964.815
137.0	9.9	27.04	4530.0	1980.0	2.288	3.92	156.239
138.0	14.38	117.86	3750.0	600.0	6.25	3.82	2772.95
139.0	17.9	29.19	8200.0	3200.0	2.562	3.82	350.5
140.0	4.29	2.42	2620.0	1120.0	2.339	4.54	5.349
141.0	8.63	105.4	7880.0	1990.0	3.96	3.5	1029.097
142.0	4.09	2.4	6550.0	2630.0	2.49	4.3	5.685
143.0	8.08	5.47	4570.0	860.0	5.314	4.03	58.279
144.0	12.18	88.9	5320.0	2870.0	1.854	3.55	565.393
145.0	5.36	11.7	6500.0	3090.0	2.104	3.85	34.265
146.0	8.39	0.42	2450.0	1880.0	1.303	4.06	1.131
147.0	15.4	7.5	3500.0	900.0	3.889	4.15	108.233
148.0	7.12	3.0	3700.0	900.0	4.111	4.66	18.844
149.0	8.58	6.0	4300.0	900.0	4.778	4.37	56.284
150.0	16.51	102.0	3400.0	900.0	3.778	4.03	1578.624
151.0	9.7	27.5	4800.0	2900.0	1.655	4.19	105.374

152.0	8.6	9.0	2600.0	900.0	2.889	4.68	47.778
153.0	6.4	50.0	5000.0	1400.0	3.571	3.41	335.149
154.0	6.1	105.0	6600.0	3100.0	2.129	3.68	370.556
155.0	5.0	63.0	2600.0	2100.0	1.238	3.59	108.635
156.0	4.7	5.7	2700.0	2500.0	1.08	4.31	6.713
157.0	4.8	79.0	4700.0	2800.0	1.679	2.7	235.746
158.0	5.5	3.7	3700.0	1700.0	2.176	4.26	10.397
159.0	15.7	1.0	9900.0	600.0	16.5	3.84	67.461
160.0	9.4	3.2	10900.0	2000.0	5.45	3.36	48.79
161.0	4.5	9.4	2500.0	1100.0	2.273	4.36	22.05
162.0	6.2	8.0	4100.0	2000.0	2.05	4.26	23.869
163.0	10.2	85.6	5900.0	1100.0	5.364	4.27	1096.744
164.0	4.4	21.0	3800.0	1700.0	2.235	4.22	48.943
165.0	9.6	1.1	1400.0	1100.0	1.273	4.47	3.007
166.0	2.2	74.6	7300.0	2000.0	3.65	4.04	148.277
167.0	6.2	15.5	2600.0	1900.0	1.368	3.91	33.633
168.0	4.4	19.1	3900.0	2000.0	1.95	4.49	36.498
169.0	8.0	21.2	5900.0	1400.0	4.214	4.39	162.812
170.0	11.3	39.8	3400.0	1000.0	3.4	3.72	411.053
171.0	14.4	34.0	2300.0	1900.0	1.211	2.3	257.684
172.0	3.1	0.6	3300.0	2300.0	1.435	4.41	0.605
173.0	5.9	4.8	1700.0	1100.0	1.545	3.75	11.671
174.0	13.0	21.2	6900.0	1000.0	6.9	4.7	404.604
175.0	8.1	14.7	6300.0	2300.0	2.739	3.82	85.379
176.0	8.6	4.9	2600.0	1100.0	2.364	4.29	23.218
177.0	7.9	55.6	5500.0	1600.0	3.438	3.38	446.712
178.0	6.9	19.7	3700.0	1000.0	3.7	3.62	138.934
179.0	9.3	12.3	6500.0	1500.0	4.333	3.98	124.545
180.0	3.4	85.5	7600.0	1400.0	5.429	3.8	415.286
181.0	4.1	61.0	5200.0	1300.0	4.0	3.39	295.103
182.0	7.3	12.3	3600.0	700.0	5.143	3.68	125.483
183.0	5.1	14.4	4100.0	1200.0	3.417	4.15	60.463
184.0	5.5	3.4	4600.0	1700.0	2.706	4.36	11.606
185.0	4.7	15.5	4100.0	1400.0	2.929	3.8	56.144
186.0	7.1	12.3	3600.0	700.0	5.143	3.68	122.045

3) Model Complexity and Event/Parameter Balance (OS)

Listwise complete cases: N=185, events=85. Parameters=7, EPV=12.1. Ridge-penalized Cox PH (λ=0.5) with z-scored covariates.

Supplementary Table S3. Cox coefficients (per 1 SD), HR and 95% CI

Variable	Coef_per1SD	SE	Z	p	HR_per1SD	HR_95%_CI
log_IPI	0.362	0.116	3.134	0.002	1.437	1.145-1.802

PDL1_Positivite	0.239	0.121	1.969	0.049	1.27	1.001-1.610
PDL1_Unknown	0.015	0.131	0.115	0.908	1.015	0.786-1.311
Age	0.21	0.114	1.836	0.066	1.233	0.986-1.542
Current_smoker	-0.245	0.136	-1.805	0.071	0.783	0.600-1.021
Brain_metastases	0.315	0.115	2.736	0.006	1.37	1.093-1.717
Therapy_line	0.059	0.106	0.555	0.579	1.061	0.861-1.306

Supplementary Table S4. Collinearity (VIF)

Variable	VIF
log_IPI	1.026
PDL1_Positivite	1.202
PDL1_Unknown	1.27
Age	1.036
Current_smoker	1.093
Brain metastases	1.101
Therapy_line	1.051

Supplementary Table S5. Proportional Hazards (approximate test)

Variable	Spearman_r_logtime	PH_p
log_IPI	-0.048	0.661
PDL1_Positive	0.388	0.0
PDL1_Unknown	-0.326	0.002
Age	0.06	0.584
Current_smoker	0.201	0.065
Brain_metastases	0.547	0.0
Therapy_line	-0.354	0.001

4) Landmark analyses at 8 and 12 weeks (OS & PFS) and Kaplan Meir Curves

Supplementary Table S6. Unadjusted Cox Hazard Ratios (High vs Low SUVmax-IPI group)

Outcome	Landmark (wk)	HR High vs Low	95% CI	p	N	Events
OS	8	2.119	1.366-3.287	0.001	181	82
PFS	8	1.224	0.795-1.884	0.359	169	93
OS	12	2.075	1.326-3.247	0.001	177	79
PFS	12	1.26	0.773-2.053	0.354	148	73

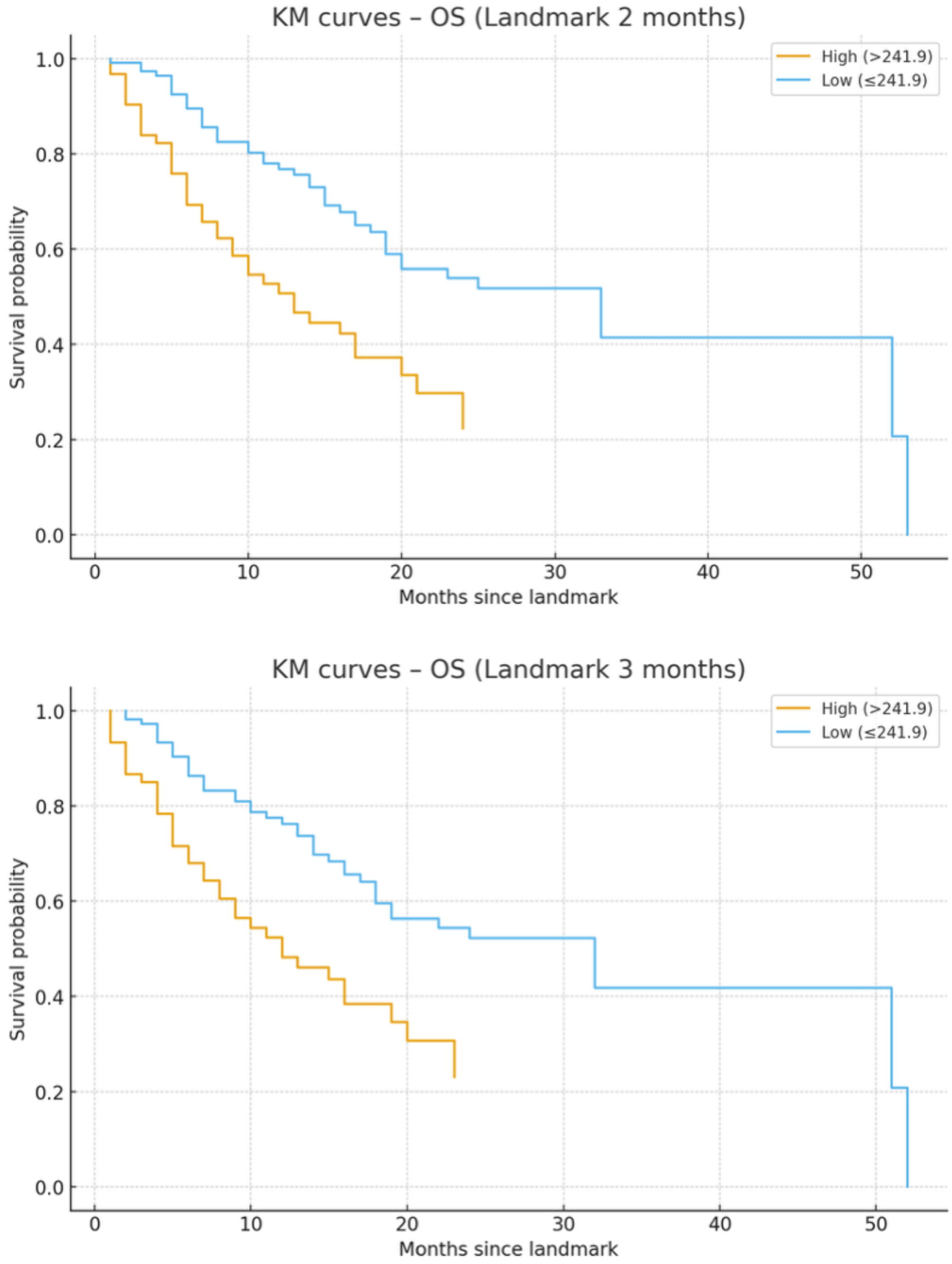

Supplementary Figure S1. Kaplan Meir Curves for OS at 8- and 12-week landmark analyses

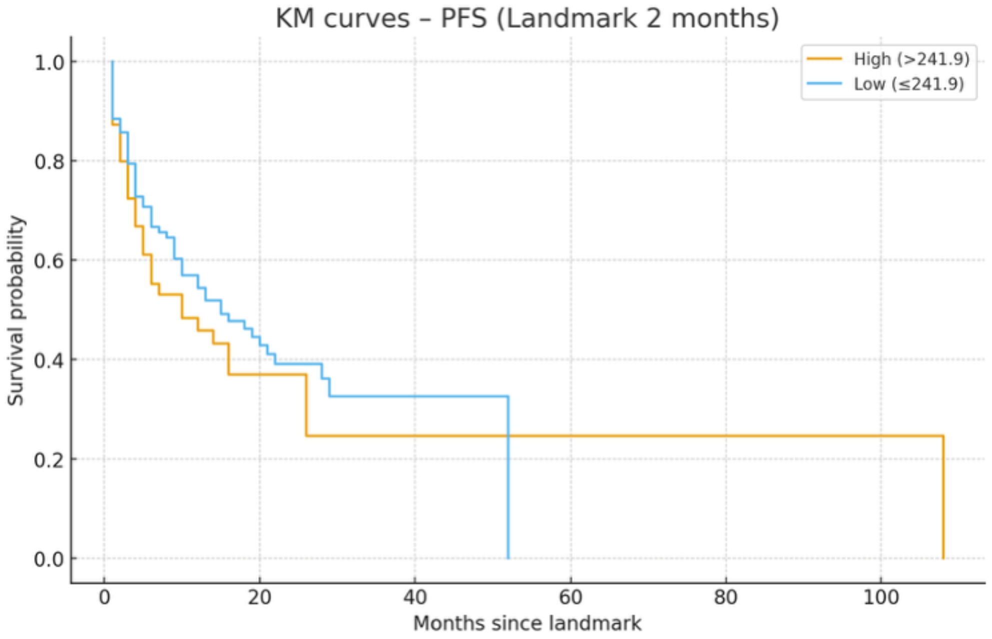

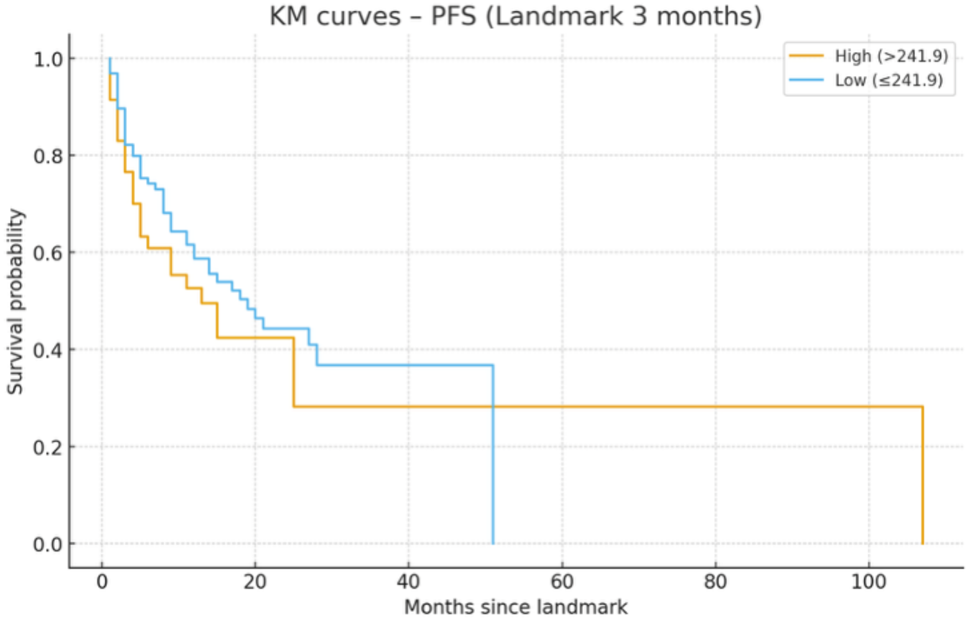

Supplementary Figure S2. Kaplan Meir Curves for PFS at 8- and 12-week landmark analyses

5) Time-dependent ROC/C-index with calibration and bootstrap

Supplementary Table S7. Time-dependent AUC at 12/24 months and C-index

Outcome	Time_mo	Metric	Value
OS	12	AUC_t	0.368
OS	24	AUC_t	0.337
OS	0	C_index	0.587
PFS	12	AUC_t	0.443
PFS	24	AUC_t	0.395
PFS	0	C_index	0.526

Supplementary Table S8. Bootstrap-corrected C-index and 95% CI

Outcome	C_index_orig	C_index_bootcorr	C_index_boot_lo	C_index_boot_hi
OS	0.587	0.583	0.523	0.651
PFS	0.526	0.525	0.472	0.599

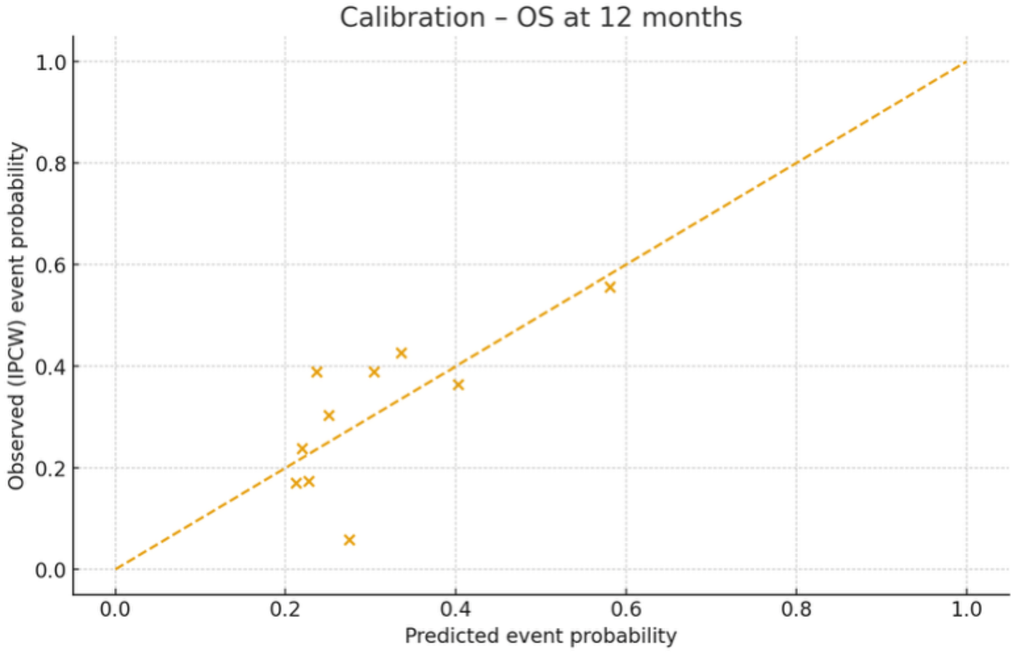

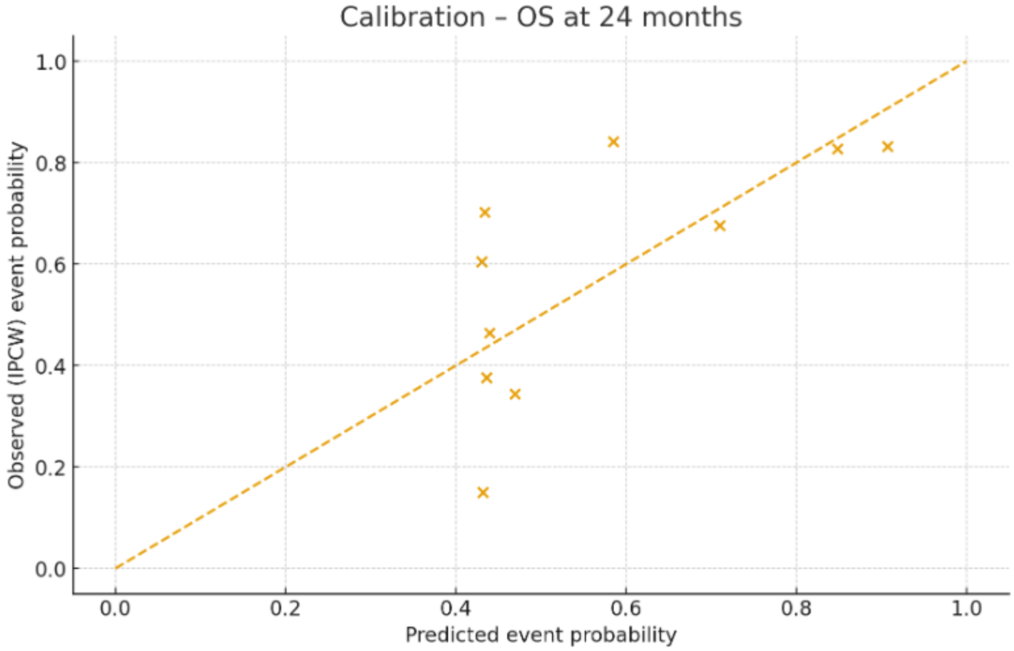

Supplementary Figure S3. Time-dependent calibration plots for OS

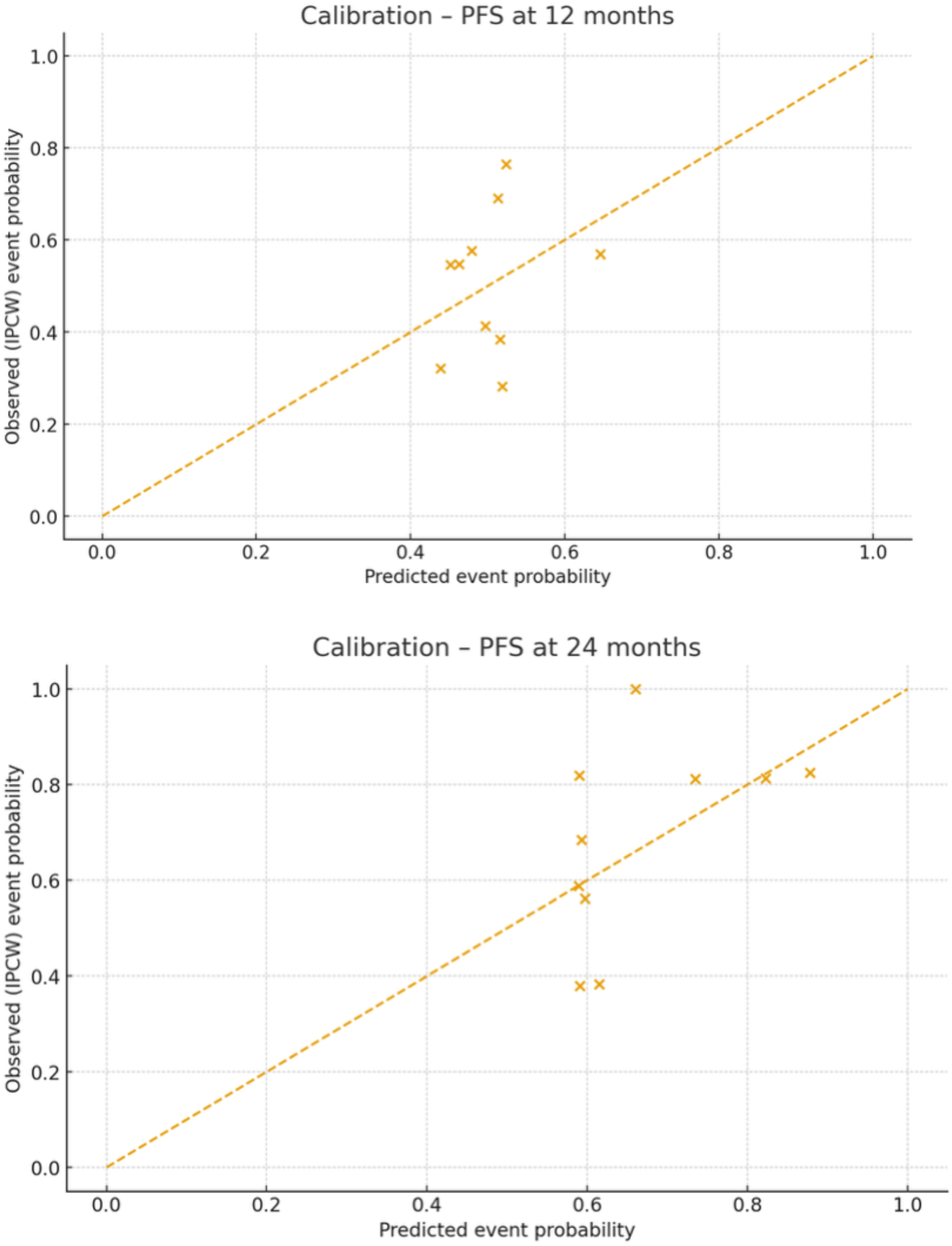

Supplementary Figure S4. Time dependent calibration plots for PFS

6) RCS effect plots for SUVmax-IPI as a continuous predictor

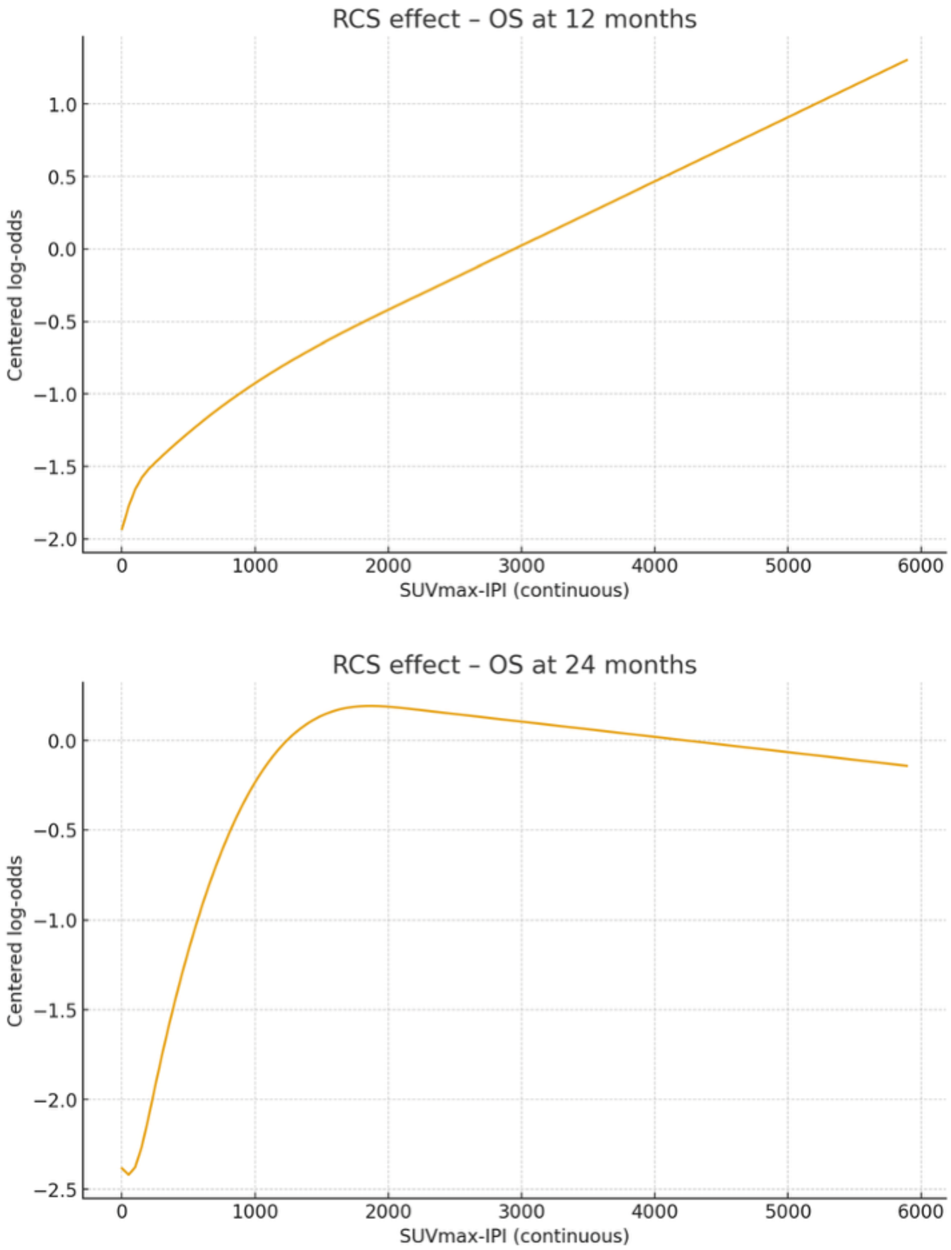

Supplementary Figure S5. RCS effect plots for SUVmaxIPI as a continuous predictor of OS

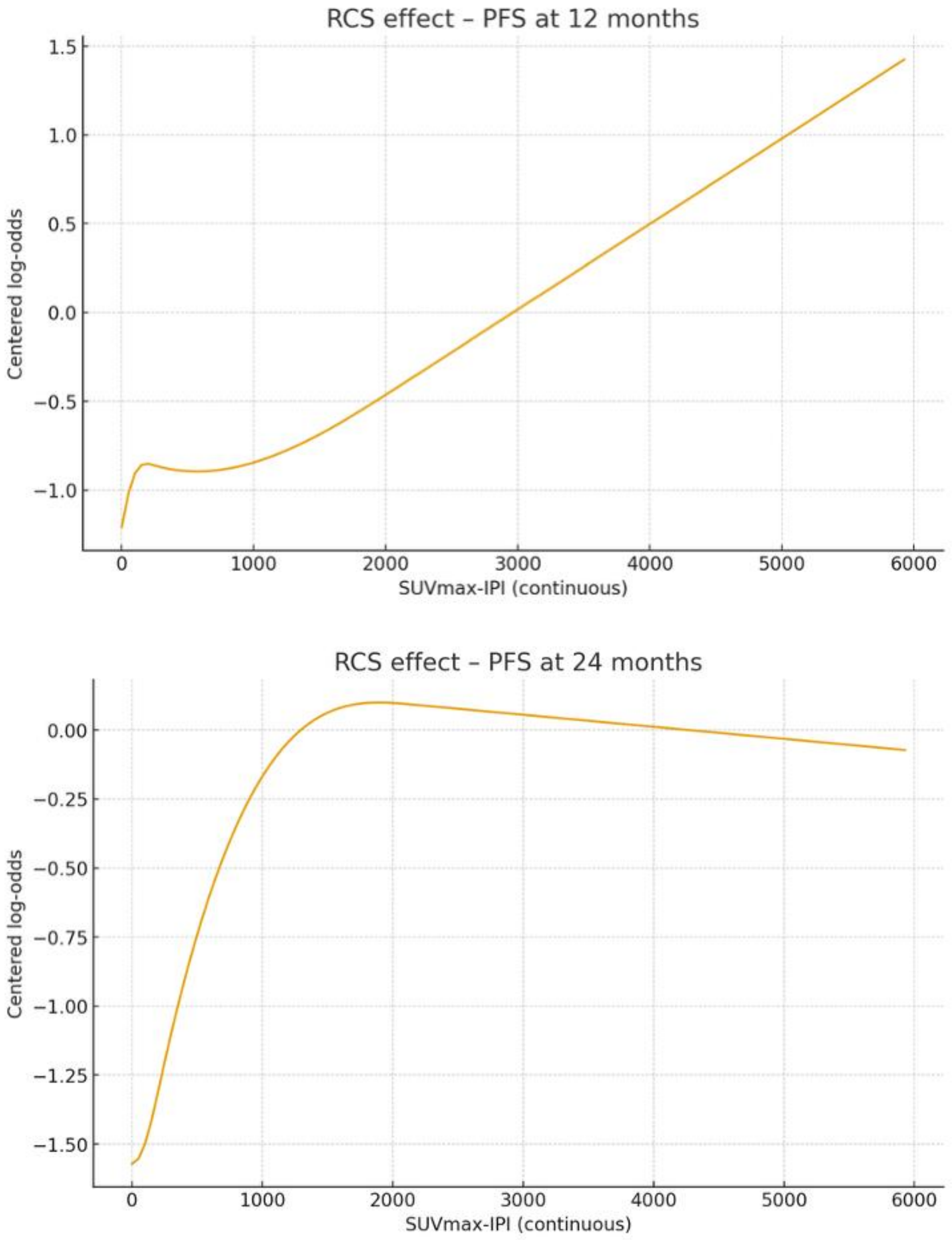

Supplementary Figure S6. RCS effect plots for SUVmaxIPI as a continuous predictor of PFS

7) Score comparison: SUVmax-IPI vs LIPI, NLR, PLR, SII and composite models

Supplementary Table S9. Score comparison for OS: SUVmax-IPI vs LIPI, NLR, PLR, SII and composite models (IPI+LIPI, IPI+NLR, IPI+PLR, IPI+SII)

Model	Time_mo	AUC_t	C_index	AIC	BIC	Delta_C_index_vs_IPI	IDI_vs_IPI	NRI_vs_IPI
IPI_RCS	12	0.368	0.587	225.427	-731.979	0.0		
IPI_RCS	24	0.339	0.587	238.408	-706.372	0.0		
LIPI	12	0.447	0.528	231.966	-728.664	-0.059	-0.048	-0.368
LIPI	24	0.455	0.528	256.907	-691.087	-0.059	-0.103	-0.651
NLR	12	0.468	0.518	231.394	-732.46	-0.069	-0.056	-0.402
NLR	24	0.412	0.518	254.999	-696.208	-0.069	-0.102	-0.608
PLR	12	0.422	0.533	229.572	-734.282	-0.054	-0.046	-0.192
PLR	24	0.423	0.533	251.699	-699.507	-0.054	-0.087	-0.508
SII	12	0.429	0.546	230.251	-733.602	-0.041	-0.05	-0.377
SII	24	0.364	0.546	252.495	-698.712	-0.041	-0.088	-0.471
IPI+LIPI	12	0.366	0.586	229.213	-721.744	-0.001	0.001	0.198
IPI+LIPI	24	0.32	0.586	242.098	-696.255	-0.001	0.001	-0.144
IPI+NLR	12	0.355	0.602	226.572	-727.61	0.015	0.003	0.212

IPI+NLR	24	0.322	0.602	240.134	-701.433	0.015	0.001	0.048
IPI+PLR	12	0.368	0.586	227.401	-726.78	-0.001	0.0	0.135
IPI+PLR	24	0.336	0.586	239.752	-701.815	-0.001	0.002	0.003
IPI+SII	12	0.358	0.597	227.217	-726.964	0.01	0.001	0.06
IPI+SII	24	0.335	0.597	240.403	-701.164	0.01	0.0	-0.267

Supplementary Table S10. Score comparison for PFS: SUVmax-IPI vs LIPI, NLR, PLR, SII and composite models (IPI+LIPI, IPI+NLR, IPI+PLR, IPI+SII)

Model	Time_mo	AUC_t	C_index	AIC	BIC	Delta_C_index_vs_IPI	IDI_vs_IPI	NRI_vs_IPI
IPI_RCS	12	0.452	0.531	259.92	-685.765	0.0		
IPI_RCS	24	0.373	0.531	233.111	-702.115	0.0		
LIPI	12	0.458	0.509	259.505	-689.394	-0.022	-0.007	-0.063
LIPI	24	0.434	0.509	231.284	-707.148	-0.022	-0.015	-0.44
NLR	12	0.514	0.508	258.764	-693.349	-0.023	-0.014	-0.155
NLR	24	0.426	0.508	233.461	-708.176	-0.023	-0.025	-0.097
PLR	12	0.51	0.504	258.572	-693.542	-0.027	-0.013	-0.141
PLR	24	0.429	0.504	231.846	-709.79	-0.027	-0.019	-0.032
SII	12	0.517	0.5	258.344	-693.769	-0.031	-0.012	-0.164

SII	24	0.374	0.5	229.074	-712.562	-0.031	-0.004	-0.001
IPI+LIPI	12	0.436	0.522	263.256	-676.001	-0.009	0.003	0.163
IPI+LIPI	24	0.349	0.522	230.659	-698.158	-0.009	0.021	-0.165
IPI+NLR	12	0.439	0.551	260.783	-681.689	0.02	0.006	0.185
IPI+NLR	24	0.372	0.551	233.531	-698.491	0.02	0.008	0.029
IPI+PLR	12	0.416	0.557	260.114	-682.358	0.025	0.009	0.14
IPI+PLR	24	0.341	0.557	231.233	-700.789	0.025	0.018	0.067
IPI+SII	12	0.424	0.553	258.788	-683.683	0.021	0.016	0.082
IPI+SII	24	0.334	0.553	230.636	-701.386	0.021	0.021	0.485

8) Decision curve analysis at 12 and 24 months

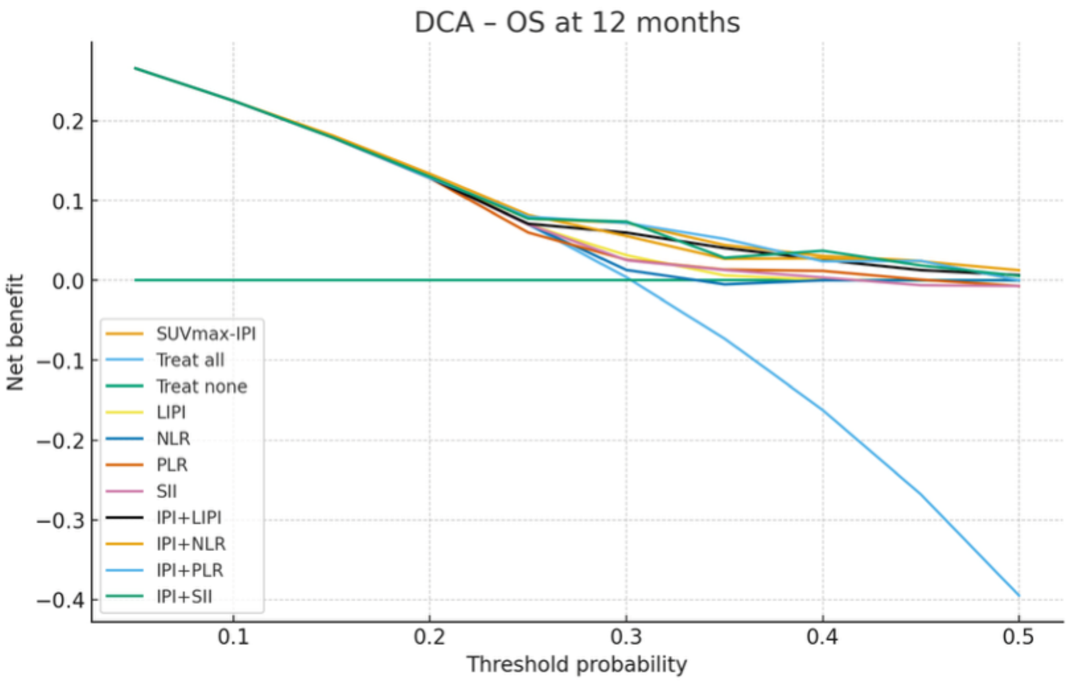

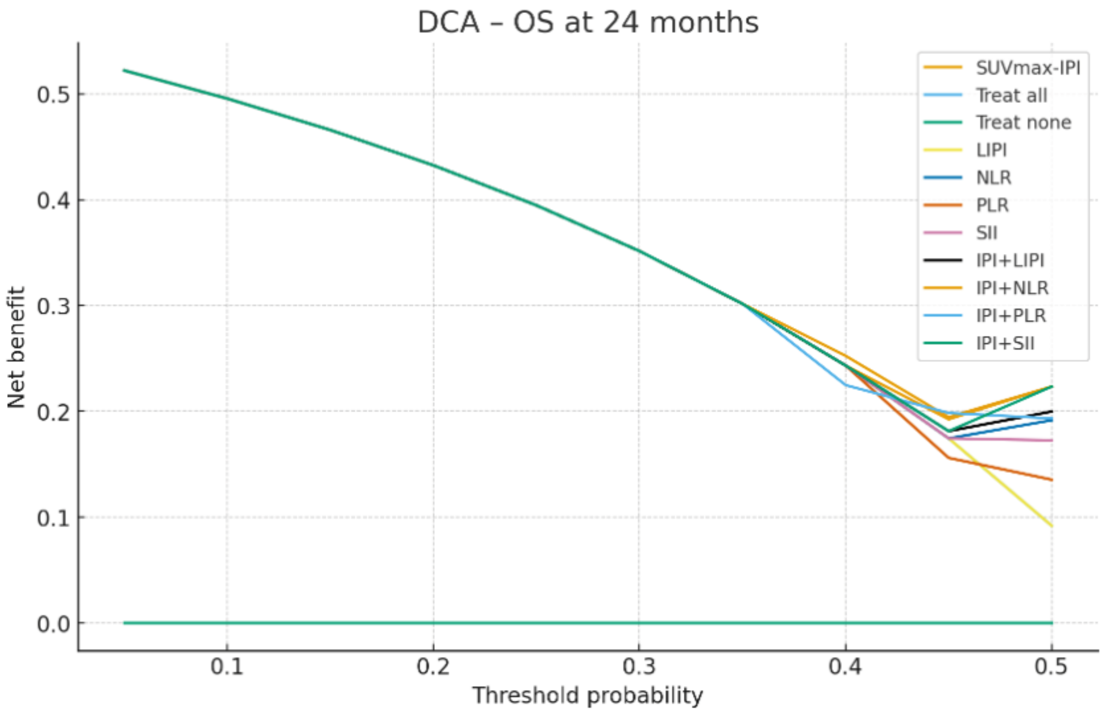

Supplementary Figure S7. Decision curve analysis for OS at 12 and 24 months

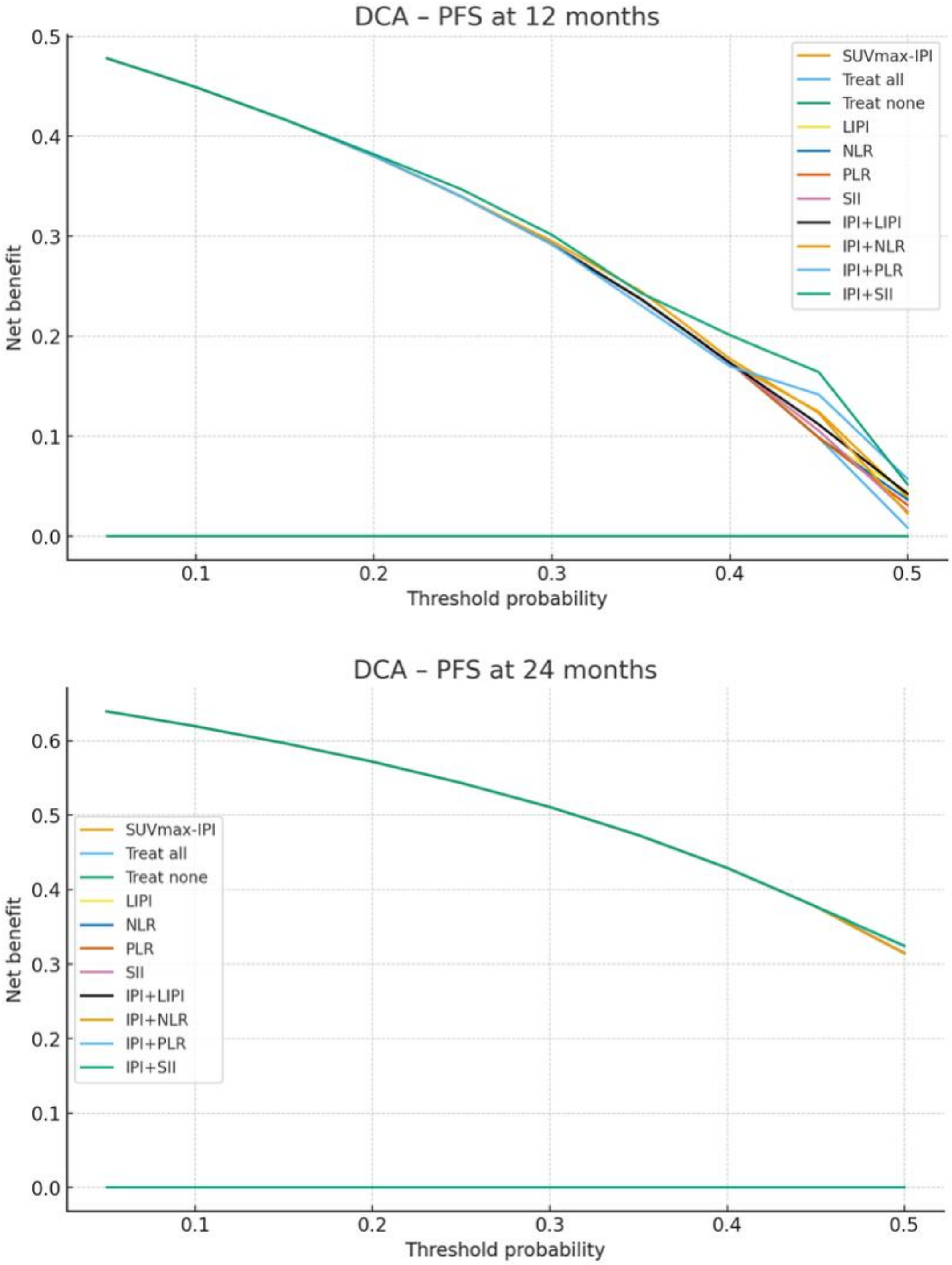

Supplementary Figure S8. Decision curve analysis for PFS at 12 and 24 months
